# Exploring Public Knowledge of Dog Law in the UK: Evidence of Poor Legal Knowledge in a Nationally Representative Sample

**DOI:** 10.3390/ani16101463

**Published:** 2026-05-10

**Authors:** Sarah A. Weir, Sharon E. Kessler, Clare P. Andrews

**Affiliations:** Department of Psychology, Faculty of Natural Sciences, University of Stirling, Stirling FK9 4LA, UK; sharon.kessler@stir.ac.uk (S.E.K.); clare.andrews@stir.ac.uk (C.P.A.)

**Keywords:** dogs, companion animals, legal knowledge, legislation, dangerous dogs, dog welfare, awareness, compliance, education

## Abstract

Dogs’ societal roles have shifted globally. In private spheres, dogs are increasingly regarded as family and granted legal protections, but in public, they are framed as dangerous and subjected to increased legal restrictions. Changing perceptions of dogs and differences in the law’s beneficiaries may influence how people develop legal knowledge. Where enforcement is limited or challenging, legal knowledge becomes an important component of the law’s capacity to shape behaviour. Using the United Kingdom (UK) as a case study, we examined people’s knowledge of 22 laws, divided between UK-wide current laws, nation-specific laws, and plausible hypothetical laws representing high-priority issues. We conducted a nationally representative survey with 1758 participants, split equally across the four UK nations. We first conducted exploratory model-building to identify variables associated with knowledge and then analysed these variables using multinomial models. Accurate legal knowledge among the UK public was limited, with participants frequently overestimating the existence of laws that prioritise dogs and their owners. Dog owners responded with greater certainty but not greater accuracy than non-dog owners. Older participants were more accurate overall, while responses to hypothetical laws suggested generational differences in views on animal welfare. These findings suggest gaps between the law in reality and what people assume the law to be.

## Simple Summary

Dogs and humans share a long and complex history, with law playing a central role in integrating dogs into human societies. When enforcement is limited, accurate public knowledge of the law can contribute to improved compliance. Recently, dogs’ societal roles have been shifting. Dogs are increasingly perceived as family in private spheres, while being regarded as dangerous pests in public, which may shape both accurate and perceived legal knowledge. Using the United Kingdom (UK) as a case study, we conducted a nationally representative UK-wide survey to examine people’s knowledge of current, nation-specific, and hypothetical laws, and the variables that influence this knowledge. Accurate legal knowledge among the UK public was limited, particularly towards hypothetical and nation-specific laws. Dog owners were more confident than non-dog owners when responding but were not more accurate. Older participants were the most confident and accurate in their responses. Younger participants were more likely to incorrectly respond as if laws exist granting additional rights to dogs and their owners, suggesting generational differences in how dogs are perceived. These findings suggest that people may imagine a more protective and developed legislative environment than currently exists, and that legal knowledge is unlikely to be playing an effective role in guiding people’s behaviour.

## 1. Introduction

Dogs are among the most popular companion animals globally, residing in approximately 12–49% of households across Europe, North America, and Australia [1,2,3,4], and ownership rates are continuing to rise in many countries that have historically had different relationships with dogs [5,6,7,8]. Their widespread presence in both domestic and public spheres means that dogs can impact those beyond their owners including people, farmed animals, and wildlife [9,10,11]. As a result, governments often use law to manage dogs’ integration into society [6,12]. Legislation is a key institutional mechanism through which governments define and enforce socially acceptable behaviour by establishing boundaries and applying punishment when those boundaries are crossed [13,14]. Law is often intended to manage and prevent conflict between stakeholders and ensure protections for specific groups, such as people, dogs, and farmed animals, in the case of dog-related laws. The effective functioning of law depends, firstly, on whether its objectives are appropriately constructed in a way that balances protections for these stakeholders in a societally acceptable manner. Secondly, it depends on the degree to which law will achieve its stated policy objectives if laws are followed by the relevant parties. However, law is also a site where the status of dogs, human interests, and ideas about social responsibility are negotiated [11,15,16,17,18]. As law is built over time, it can retain and embed some beliefs that may now conflict with current or evolving social norms or scientific evidence [19,20,21]. Therefore, what it means for law to ‘function effectively’ can change over time, and even compliance with current laws may not necessarily lead to positive outcomes for all stakeholders.

The third key aspect of effective legislation is the degree to which targeted groups comply with the laws applicable to them. This compliance can be achieved through enforcement and punishment of those who cross the stated boundaries of behaviour (post ante function of law) and also through shaping people’s behaviour before rules are broken, known as the ex ante function of law [22]. The reliance on law’s ex ante function may be increasingly important in the functioning of dog-related law because enforcement agencies are facing a range of challenges in enforcing dog-related legislation effectively. Some of these challenges are a result of the resourcing and complexity of the agencies involved in enforcement. These can include lack of funding, appropriate training, the high costs of kennelling affected dogs, and the complex number of agencies involved, both government and non-governmental, which can also vary between issues and within a country [23,24,25,26,27,28,29]. The law itself can make enforcement more challenging. Some laws, such as licencing, cruelty offences, and certain dangerous dog provisions, have been described as difficult to enforce due to challenges in detecting offences and ambiguity in the laws’ language defining when an offence has occurred [23,25,26,29,30,31]. As consistent and predictable enforcement can be an important aspect of increasing compliance [22], these challenges place greater emphasis on the functioning of law in shaping behaviour before violations have occurred.

Using law to pre-emptively shape behaviour can be challenging because compliance is complex. Theoretically, the route to compliance is linear: governments publish laws, people learn them and understand their new legal responsibilities, and then decide whether to follow them [13,32]. In practice, compliance is more complicated than this and can be shaped by the degree to which law aligns with social norms, the capacity and opportunity someone has to comply or offend, the predictability of enforcement, people’s degree of trust in institutions, and people choosing to comply after weighing up the costs and benefits of doing so [33]. Running through many of these theoretical ideas is the assumption that people have sufficient knowledge of the law. For example, it argued that for people to have sufficient capacity to comply or make a rational decision, they must have adequate knowledge of what is expected of them as a prerequisite [13,32,33]. In the context of dogs, Tadich et al. [34] found that those who self-reported as less aware of Chile’s animal welfare and dog legislation had reduced odds of reporting that they performed the required behaviours, such as licencing and vaccinations. This evidence, while only correlational, is at least consistent with a link between knowledge of law and compliance. Regardless, the importance of legal knowledge is also reflected in the legal system itself. As ignorance of the law is generally not accepted as a defence, legal systems place the onus on individuals to ensure they are aware of their legal obligations [13]. Therefore, legal knowledge is a core aspect in promoting voluntary compliance, albeit not necessarily by itself sufficient for compliance nor linear in its effect [32].

Legal knowledge may be particularly important in the context of dog-related legislation. During the twentieth century, there was a shift from regulating dogs directly (e.g., seizing out-of-control dogs in public spaces) to regulating their owners, who are held individually responsible for the conduct of their dogs (e.g., making a person guilty of an offence for a dog being out of control in public) [6,30]. This shift has individualised responsibility, resulting in dog owners being responsible for negotiating various societal interests through their everyday interactions [6,17,30]. As a result, owners have become central to the operation of legislation, as compliance and enforcement depend on their behaviour and decision-making. In this context, legal knowledge is likely to become particularly important. People’s knowledge, or perceptions of what the law is, may shape how individuals interpret dog-owning responsibilities and influence how law is enacted and socially negotiated in everyday settings.

Despite this importance, research suggests that legal knowledge across law areas is limited [32,35,36,37,38] and findings from non-animal-related law demonstrate that the public’s perceptions of what the law is can be shaped by social attitudes and norms [32]. In the absence of sufficient knowledge, people may rely on their normative instincts (their internalised ideas of right and wrong) to make assumptions about what legal rules exist [13,32]. Attitudes towards dogs vary [39,40,41,42], as do attitudes towards how dogs should be integrated into public and private spaces [15,16,43,44,45,46]. This variation may lead people to both correctly infer existing dog-related laws and to develop false assumptions about the extent of legal protections afforded to dogs or their owners.

These differences in attitudes reflect, in part, the shifting role and status of dogs globally. While owned dogs were previously kept mainly for work or status, they are now increasingly kept primarily for companionship and are becoming integrated into more-than-human families [47,48,49,50,51,52]. This shift is reflected in the language used by people to describe their dogs. Increasingly, dogs are described as family, children, or friends, suggesting their increased perceived humanisation [49,53,54]. (The language used by academics is also changing, and terms such as ‘dog owner’ are increasingly challenged, sometimes being replaced with alternatives such as ‘dog guardian’ [55]. In this paper, we use the term ‘owner’ to refer to individuals who live in a household with companion dogs because it is the term often used in law and by Western governments.)

Concurrently, dogs have also been increasingly controlled in public spaces because of fears that they may cause injury, spread disease, and cause environmental and neighbourhood nuisances [24,56,57,58]. Compounding these complicated and shifting perceptions of dogs is the fact that companion dogs legally remain personal property of people [59,60,61]. These changes have increased expectations of dogs’ behaviour, while also making them entirely reliant on their owners to meet their needs, in part because in many countries dogs are no longer permitted to roam freely and can no longer access public space independently of their owners [15,49,62,63,64]. These changes may result in people forming different assumptions of what the law is based on their experiences of dogs and perceptions of dogs’ status.

These complex and often contradictory views of dogs can be described as dogs now living separate public and private lives [6,12,15], which may be differently legislated [65]. The ‘public dog’ is a dangerous, annoying property that needs to be controlled [12,15,64]. The ‘private dog’ is a family member deserving of the same care and protection as other human family yet subject to their owners’ demands [63,66,67]. Weir et al. [65] found evidence for this divide in how dog-related law benefits stakeholders in the UK. In public, the interests of the general public were overwhelmingly benefited while dogs were not benefited. In contrast, in private, dogs benefited, largely from animal protection legislation, while the general public was not impacted. Dog owners received few benefits from the law and were faced with substantial legal obligations. Thus, in the UK at least, there is a distinction in the legislation of dogs’ private and public lives which may impact the daily lives of dogs and their owners.

These distinctions in how the law benefits stakeholders, coupled with the complex and changing status of dogs, may shape how legal knowledge is formed and understood. The volume and complexity of law, both generally and within animal law, has increased [22,68], which may make it increasingly challenging for dog owners to know all of their legal obligations [28]. Instead, they may need to prioritise the knowledge they acquire. Owners receive few benefits from the law [65] and sometimes prioritise their dogs over others’ interests, including people, other dogs, and wildlife [69,70,71,72]. When the law does not appear to align with the interests of dogs or their owners, dog owners may find it more difficult to rely on their normative intuitions when estimating their legal requirements and may overestimate the legal protections afforded to themselves or their dogs [32]. In a similar fashion, non-dog owners may pay closer attention to laws perceived to protect public safety or their own interests. These dynamics may result in dog owners and non-dog owners holding different understandings of the law, with implications for conflict, the reporting of perceived wrongdoing, and levels of institutional trust.

### 1.1. Implications of Divergent and Poor Legal Knowledge Between Stakeholders

Different levels of legal knowledge between dog owners and non-owners may result in different expectations of behaviour and, when those expectations are not met, conflict may occur. The potential for tensions may be growing due to different expectations of dog behaviour between dog owners and other community members. Dog owners can underestimate the negative impacts their dogs can have on others [69,73,74,75]. This is at a time when people’s expectations of dog behaviour are rising, increasingly to unrealistic levels, with dogs often expected to behave in line with what is expected of adult humans [6,15]. These divergent expectations of what acceptable behaviour is may lead dog owners and other community members to form different assumptions of what current legislation is, potentially causing frustration and conflict. For example, Eldrige and Jović [17] found that dog owners’ access to public transit in London, UK, was frequently a source of conflict due to an incorrect belief by the driver and passengers that access was dependent on the driver’s discretion, causing dog owners to be frequently denied access. As the dog owners interviewed often knew that their dog should be allowed according to the transit system’s rules, their dogs’ access was often negotiated, resulting in a range of experiences of the service. Together, these dynamics illustrate how uneven legal knowledge and shifting social expectations of dogs have the potential to play a role in undermining law’s capacity to prevent conflict and instead might even exacerbate it.

In contrast, in the private sphere, non-compliance with law by dog owners is often invisible to formal enforcement mechanisms, making third-party reporting essential for bringing potential unlawful acts to the attention of the relevant enforcement agencies [23]. However, effective reporting requires community members to have appropriate knowledge of the legal boundaries of animal cruelty or welfare offences. Government and academic sources have highlighted how limited awareness of animal welfare laws is limiting their effectiveness [23,26,76]. This lack of awareness may result in people being unable to effectively recognise and report instances of unlawful animal cruelty or poor welfare. For example, Glanville et al. [77] found in Australia that 27% of participants surveyed took no action after witnessing cruelty or neglect, with the most common reason being uncertainty about whether mistreatment was truly taking place. Therefore, appropriate legal knowledge is not only important for guiding people’s behaviour but also for improving the public’s ability to support enforcement agencies in detecting wrongdoing. Consequently, it is vital for community members to possess sufficient knowledge of what constitutes a welfare or cruelty offence in order to accurately recognise and report these to the relevant authorities.

Additionally, the ex ante function of law may be further limited by dog owners themselves being unsure about the threshold at which poor practice becomes a welfare offence, reducing the effectiveness of the ex ante function of law. Laws that aim to protect dog welfare are often for all owned animals and can use ‘vague’ language, such as ‘unnecessary suffering’, which requires court judgements to interpret and apply to individual cases [78,79]. Governments frequently rely on codes of practice to provide species-specific guidance to animal owners [78,80]. However, a post-legislative review of the Animal Welfare Act 2006 in England and Wales concluded that England’s Code of Practice had not effectively communicated legal responsibilities to animal owners, despite enabling a wider range of prosecutions [76]. This suggests that the Act performs more strongly as an enforcement tool than as a mechanism for guiding behaviour in advance.

Dog owners may require clearer and more prescriptive legal guidelines to ensure they meet their dogs’ welfare needs and enable the law to have a stronger ex ante function. Large-scale UK [81,82,83] and Australian studies [1,84,85] report wide variation in dog owners’ knowledge, attitudes, and behaviours related to their dogs’ care and welfare. Part of this variation is a result of dog owners’ beliefs in what are good or ‘responsible’ practices [71,83,85]. For example, Rohlf et al. [85] found that although all dog owners surveyed agreed that dog walking was good for their dog’s health, 95% of participants reported that their dogs received ‘adequate’ exercise, yet only 60% reported exercising their dogs seven times a week. This difference in reported beliefs, perceptions of their behaviour, and actual behaviour may result from a lack of specificity of guidance. For example, the UK’s codes of practice vary across the country. England’s codes of practice recommend ‘regular’ exercise, Wales and Scotland recommend ‘suitable’ exercise, while Northern Ireland recommends at least daily exercise [86,87,88,89]. This variability in guidance may allow owners to interpret welfare guidelines according to their own beliefs, potentially contributing to inconsistent practices and limiting the preventative function of law.

To address these challenges, some countries are creating more defined legal limits of behaviour to protect dog welfare. In a comparison of dog welfare laws of culturally Western countries, Andersen et al. [80] found that the scope and specificity of dog welfare legislation differed substantially. While some countries such as Australia, New Zealand, and England were more likely to use non-binding guidelines, others such as Germany and Sweden created more specific legislation, governing the day-to-day lives of dogs and their owners. Ministers in countries that introduced more specific legislation have increasingly referenced the sentience and family status of dogs [90,91,92]. This suggests that as dogs’ roles change, some countries are beginning to adapt by using the law to signify their importance and elevated role. It is currently unknown whether people in countries that tend to use more non-binding guidelines, but whose perceptions of dogs are shifting, assume that the law provides greater protections for dogs than it actually does.

### 1.2. Demographic Variables Influencing Legal Knowledge

Despite the importance of legal knowledge for voluntary compliance, third-party reporting, and the management of conflict between stakeholders, current evidence concerning the public’s knowledge of dog law is limited. There are two key limitations, which our present study seeks to address. First, previous studies have found mixed results on the differences in knowledge of law between dog owners and non-owners when the general public was targeted [35,36,38,93]. Four studies targeted both dog owners and non-owners and three of these found no evidence of dog ownership status influencing the knowledge or awareness of dog law [35,36,93]. Only Weng et al. [38] found a significant difference between these groups; however, both groups scored below the study’s threshold for inadequate knowledge of 80%, and only 24% of the sample were dog owners. It is possible that no effects were found because the non-dog-owning group could consist of people who had previously owned a dog and had a childhood dog. These experiences may result in differences in knowledge because of their past experiences and continued interest in dog-related topics [94], which could obscure any differences between dog owners and non-dog owners. Additionally, many of the studies used convenience samples and so may have recruited people who are more interested in dogs, regardless of ownership status. Therefore, it may be important for studies to consider more in-depth experiences of dog ownership when testing people’s legal knowledge of dog-related laws.

Secondly, results are similarly mixed about other variables that may influence knowledge of dog laws. To our knowledge, only eight studies have investigated either self-reported awareness of law (e.g., how aware are you of the Animal Welfare Act?) [34,81,93] or tested participants’ knowledge [35,36,37,38,95]. The studies that tested people’s knowledge found it to be generally inadequate, but that knowledge level could vary by topic. Self-reported awareness of law varied but was relatively higher than when knowledge was tested, suggesting that people think they know the law better than they do. The demographic variables associated with knowledge or awareness were inconsistent across these studies. Some studies found variables like age [37], gender [38], location such as cities [93] or subnational jurisdictions [81], education [37,38], or occupation [37] influenced knowledge, while other studies found no significant differences for these effects [35,36,37]. For example, Weng et al. [38] found that men answered questions more accurately than women, while Keogh et al. [36] found no difference between genders.

These mixed results are likely a result of the sampling methods used. All studies so far have used convenience samples or snowball sampling, or have recruited at specific locations such as universities [36] or schools [38]. This resulted in many of the studies having overrepresented groups such as women [34,35,36,38] and likely introduced self-selection bias towards people invested in dogs. These imbalances may have made it more challenging to detect effects and may have limited the generalisability of the findings to the wider population. Building on this prior research, the use of a nationally representative sample testing participants’ knowledge of dog law would be beneficial in strengthening the robustness and generalisability of findings across diverse population groups.

### 1.3. The Present Study: An Examination of People’s Knowledge of Current, Nation-Specific, and Hypothetical Laws, and an Exploration of Demographic Variables That Influence This Knowledge

The United Kingdom (UK) provides a useful context for addressing these research gaps. Dogs are popular in the UK, with 36% of households owning at least one dog [4], and there is a long tradition of legislating dogs [58,68,96]. Additionally, the distinct approaches of increased restrictions on the ‘public dog’ and increased protections for the ‘private dog’ have their roots in Victorian England [68,96,97]. These approaches continue to influence current UK law, whereby laws governing the ‘public dog’ overwhelmingly prioritise people, while laws governing the ‘private dog’ prioritise dogs [65]. England was also found to be a country that has little prescriptive animal welfare law [80] but has a population that is supportive of increased animal welfare legislation [98]. This may make it an appropriate place to investigate the public’s assumptions about more prescriptive laws that are not in force in the UK but have been proposed or are in force in other countries.

The UK’s political landscape also enables comparison between different UK nations to test whether local nation-specific laws are better known than UK-wide laws. Testing participants’ knowledge of laws that are in force in only one part of a country has been used to assess whether the knowledge component of the ex ante function of law operates effectively [13]. The UK is a unitary system with partly autonomous (known as devolved) nations that vary politically, demographically, and culturally [99]. The devolved structure of the UK has created a system in which the four nations, England, Scotland, Wales, and Northern Ireland, can each enact animal-related legislation. Consequently, some laws are in force UK-wide, others pursue similar objectives through different provisions, and some exist only in a single nation. Other studies provide an indication that self-reported awareness of law can differ across areas within a country [81,93], including studies focusing on non-animal law [32]. Therefore, the UK provides an interesting context to examine people’s knowledge of the laws in their specific nation, in the country as a whole, or whether they perhaps incorrectly respond as if laws exist that provide greater protections than they actually do in reality. This study includes laws in force throughout the UK (current laws), nation-specific laws, and more prescriptive laws not currently in force in the UK, based on the phrasing of real European legislation where possible (hypothetical laws).

Taken together, this study builds on our earlier work [65] in several important ways. This study examines the UK public’s knowledge of current dog-related legislation, explores how this knowledge may vary across the UK, and assesses whether people assume that the law provides more specific protections for dogs and owners than are currently in place, potentially reflecting dogs’ changing position in society. By using an exploratory model-building approach, we also explore the variables that may shape this knowledge to gain a deeper understanding of the development of knowledge. We used an exploratory model-building approach instead of testing specific hypotheses because past research has found conflicting results concerning demographic sources of variation in law knowledge and thus could not guide defensible hypothesis formation. This study therefore aims to provide a basis for subsequent, hypothesis-focused research [100]. It draws on a UK sample evenly distributed across the four nations and representative of the UK’s population by age and gender and a research design that exploits variation in the expected familiarity of laws across locations.

## 2. Materials and Methods

### 2.1. Ethical Approval

This study received ethical approval from the General University Ethics Panel (Reference: GUEP 2024 15873 14069) and the Animal Welfare and Ethical Review Body (Reference: AWERB 2023 15697 11124) at the University of Stirling on 23 June 2024.

### 2.2. Recruitment Process

Cint, a market research company, was employed to survey a nationally representative sample using their network of survey panels. To ensure sufficient representation of the four nations, we aimed for our sample to be split evenly between Northern Ireland, Scotland, England, and Wales. These participants were then nationally representative of the UK as a whole based on age and gender. England’s participants were additionally representative of the nine English regions defined by the Office of National Statistics (e.g., East Midlands, South East, London, etc.) [101]. We used interlocking quotas where participants must meet multiple criteria (e.g., we aimed to recruit 25 participants who were Welsh, identified as female, and were aged between 18 and 24) [102]. Participants were eligible for the survey if they permanently resided in the UK, were over the age of 18, and could complete the survey in English.

Data collection ran between 31 July 2024 and 11 August 2024. The online survey was deployed to Cint’s research panels, and interested panellists could decide to participate after giving their informed consent. A total of 2970 panellists accessed the survey with a 60% completion rate, resulting in 1758 participants completing the survey. In total, 744 participants were rejected because they did not meet the eligibility criteria (65), a quota was already filled (597), or they failed attention checks (82). To pass the attention check, participants had to choose yellow from a list of nine colours and correctly solve a simple maths problem. A further 468 participants were rejected because they exceeded the time limit of 1 h for an estimated 10 min survey.

This process resulted in 1758 participants, 95% of whom had complete data. An option of ‘prefer not to say’ was offered for gender, income, and education. Sixty-seven participants chose ‘prefer not to say’ for income, which was the most frequently unanswered question. Nine participants chose ‘prefer not to say’ for education, seven of whom also did not choose an option for income. Two participants chose ‘prefer not to say’ for their gender. An additional three participants were non-binary or chose the ‘other gender identity—please specify’ option to describe their gender identity, which we set to missing due to the small numbers. Twelve participants entered a postcode that did not match their selected country, which resulted in missing data for both UK Nation and Urbanicity (details below). There was no evidence of a pattern in the demographic characteristics of these participants with missing data or evidence that they responded differently to the other participants (see File S1 for further details and results from analysis conducted using the VIM package (version 6.2.2) [103] in R using Version 4.4.2).

To maximise the use of participants’ data, we created two datasets, one where missing data was input as NA (named the Not Available dataset and hereafter described as the NA dataset) and another where participants were removed if there was any missing or incorrect data (Removed Missing dataset, hereafter referred to as the RM dataset). This process resulted in 1673 participants in the RM dataset. Candidate models were compared and selected using the RM dataset to ensure results were not due to differences in the number of participants included across models. Once a model was selected using the RM dataset, the model was rerun using the NA dataset, and these results were reported. As a result, the number of participants differs in these final models.

### 2.3. Demographic Variables

Participants were first asked about demographic variables including age, gender, income, education, their UK nation of residence, and the first half of their postcode to identify if they lived in a predominantly urban or rural area (see below). Participants were then asked about their dog ownership status and previous dog ownership (hereafter ‘Dog History’) and whether they had a dog in their home as a child (‘Childhood Dog’). See File S2 for the full survey.

To create a more detailed understanding of a participant’s history with dogs, participants were given multiple options and could select more than one. These options included ‘currently own at least one dog’, ‘previously owned a dog as an adult’, ‘had a dog in the household as a child’, and ‘never owned a dog’. From these responses, we created four groups: ‘First-Time Owners’, ‘Longtime Owners’, ‘Past Owners’, and ‘Never Owners’. These groups are defined in Table 1. However, the ‘Longtime Owner’ group had only 82 participants (4.7% of all participants), and so a second variable was created, ‘Current Owner’, which merged this group with First-Time Owners. During exploratory model building (see Section 2.7), we compared the performance of models using ‘Current Owners’ and ‘First-Time Owners’ as reference categories. Results will specify which reference category was used. A separate binary variable was created called Childhood Dog based on whether someone selected ‘had a dog in the household as a child’ (1) or not (0).

The variable that measured the extent to which a participant’s area is urban or rural was developed using the relevant UK countries’ classification system [104,105,106] in reference to the first half of the participants’ postcode (postcode district). We only collected the postcode district to maintain participant anonymity. As urban/rural specifications are conducted at the granular postcode/settlement level, we created a percentage of how urban the postcode district was (Urbanicity). A score of 0% indicates that a participant lives in an area where every postcode district is classified as rural, while a score of 100% indicates that all postcode districts are urban. See Appendix A for a full discussion of how we calculated an Urbanicity score for each UK nation and File S3 for the data used to calculate these scores.

### 2.4. Law Item Development

We developed a questionnaire that included laws currently in force in the UK (current laws), laws only in force in one of the four UK nations (nation-specific laws), and laws not in force anywhere in the UK (hypothetical laws). Due to budget constraints and to reduce the risk of participant fatigue by keeping the survey within approximately 10 min, we included 22 law items across all categories (see Table 2).

Laws were chosen based on the priority of the issue for stakeholders and welfare issues for UK dogs, how detailed the law’s requirements were, and whether the item could be classified into either a law that governs the public or private dog to include laws that prioritise different stakeholders. To operationalise the idea of the ‘public dog’ from Carter, and reflecting the ideas from Srinivasan [64], Instone and Sweeney [15], and Howell [97], we defined a law as ‘public’ when a dog impacts, or has the potential to impact, someone outside of the family. While this approach operationalises Carter’s framework narrowly, our aim was to classify laws with consistency, while also taking into account the ways in which dogs can become legal subjects through both perceived and real risks. For instance, although a law that says it is an offence to cause a person to be reasonably afraid of being attacked in private is regulating an event occurring in the domestic sphere, we categorised this as a public law because the dog’s interactions with people outside of the owner’s family are being legislated. We excluded all laws that involved the breeding and sale of dogs because they mostly focus on the actions of those involved in selling or breeding dogs rather than most dog owners or the general public.

#### 2.4.1. Current and Nation-Specific Laws

We created an initial list of 11 current laws and eight nation-specific laws from Keogh et al. [36] and Weir et al. [65]. Of the eight laws from Keogh et al. [36], five of these related to governing the public dog. We excluded two of the three provisions that protected dogs. We excluded Keogh et al.’s [36] provision, ‘All owners must keep their animal in a way that safeguards its health and welfare’, because it did not include detailed requirements and people may interpret what behaviours are required to meet this statement differently [71,81]. Additionally, we excluded a provision about the requirement of a veterinary surgeon to dock dogs’ tails because it is often breeders who dock a dog’s tail within the first week of birth [108]. Additionally, it is the availability of exemptions for docking working dog breeds tails that is debated in the UK [109]. We therefore included the ban on ear cropping to represent the UK’s mutilation ban because there are reports that this practice is increasing, with members of the public able to buy ear cropping kits that can be used at home [110].

Our initial list was dominated by laws governing the public dog. To identify additional dog welfare laws that met our inclusion criteria, we checked the dog protection sections of law identified in Weir et al. [65] that excluded breeding and sale of dogs. Of the 33 relevant sections, only four sections met our criteria, one of which was already in our list as a result of it being included in Keogh et al. [36]. These were the nation-specific laws of banning electronic collars (also known as e-collars or shock collars) in Wales and the provision in Scotland that says government inspectors can transfer ownership without the need for a court order due to a dog suffering unnecessarily. The remaining provision was banning mutilation, which we included as a ban on ear cropping. This resulted in eight current UK-wide laws being included and four nation-specific laws. See File S4 for all laws identified and for the reasons for their inclusion.

#### 2.4.2. Hypothetical Laws

We included ten hypothetical laws from an initial list of 17 laws. Twelve of these laws were identified using the supplementary materials from Andersen et al. [80] and laws that were of high priority to UK dog charities, governments, and parliamentary and multi-stakeholder policy groups. We identified an additional five laws by reviewing the campaign pages of eleven dog charities and government pages from different UK nations in June 2024 (see File S4 for a list of sources and the process to identify relevant issues). We excluded seven laws from our initial list because they were not included as high priority to UK charities and governments or were legislating the breeding and sale of dogs. We based the phrasing of the hypothetical laws on real European countries’ legislation where possible to ensure their plausibility and prevent participants from identifying them as incorrect based on phrasing alone. All items were designed to mirror the phrasing and structure of Keogh et al. [36] to maintain consistency. See File S4 for all laws identified and for the reasons for their inclusion.

### 2.5. Data Analysis

We used an exploratory model-building approach (see below for more details) and ran all statistical analyses in R [111] using Version 4.4.2 and used Chat GPT (primarily the GPT-4o model) selectively to support troubleshooting coding issues and generate initial code templates. These were reviewed and adapted before being incorporated into the analysis. See Files S5 and S6 for codes used to prepare and analyse the data. We fitted a separate model for each of the 22 law items to account for the variation in item topics, the status of law in the UK, and to ensure the results would be relevant and useful to stakeholders working in these specific areas. We tested the correlations between items using Cramer’s V because the outcome variable is unordered [112]. Scores varied between 0.11 (weak correlation) and 0.37 (moderate correlation). Correlations between independent variables were calculated using the relevant statistics based on the variable’s measurement scale (ordinal, nominal, binary, etc.) [112,113,114,115,116,117,118,119]. Using the relevant interpretation for each statistic, we found that two combinations (Age and Dog History, and Income and Education) had a moderate association. Dog History and Childhood Dog was the only combination that had a relatively strong association (Cramer’s V = 0.4). No independent variables had strong correlations. See File S7 for all correlation results, the tests used, and their interpretation.

To measure participants’ knowledge of dog law, we created a variable ‘Correctness’ from their responses of “True”, “False”, and “Don’t Know”. As four items are only in force in some parts of the UK, this variable took a participant’s country of residence into account. For instance, if an English and a Welsh participant both chose ‘True’ when answering Shock Collar Ban, which is only true in Wales, the English participant would be coded as ‘Incorrect’, while the Welsh participant would be coded as ‘Correct’. To analyse these responses, we used multinomial analysis, which compares categories j (Don’t Know or Incorrect) to a baseline J (Correct) using the ‘nnet’ package (version 7.3.19) [120]. The multinomial logit model is defined as

(1) $$log\frac{P\left(Y=j\right|x)}{P\left(Y=J\right|x)}=\alpha_{j}+\beta_{j}x_{1}+\beta_{j}x_{2},\;where\;j=1,\ldots ,J-1$$

Y: the unordered outcome category;

*J*: the baseline category (Correct);

j: specific category other than the baseline (Don’t Know or Incorrect);

$\alpha_{j}$: threshold intercept for category j;

x: predictor values;

$\beta_{n}$: coefficient for predictor x.

### 2.6. Interpretation of Adjusted Odds Ratios

Multinomial models are a type of logistic regression which produce adjusted odds ratios (AORs). AORs are an indicator of change in the odds of the outcome resulting from a change in the predictor while accounting for the effects of other variables in the model [119]. Odds are calculated as the probability of an event occurring divided by the probability of the event not occurring [119]. The AOR is then created by calculating the proportional change in odds between two categories [113]. Adjusted odds ratios represent the odds of the outcome occurring relative to the predictor’s baseline category, holding all other variables in the model constant. A value of 1 indicates equal odds, values greater than 1 indicate higher odds, and values less than 1 indicate lower odds. Additionally, to aid interpretation, adjusted odds ratios between −2 and 2 are referred to in percentages. For example, an adjusted odds ratio of 1.5 for Never Owners indicates a 50% increase in the odds of being incorrect relative to its reference group, Current Owners.

### 2.7. Variable and Model Selection

We used an exploratory model-building approach because there are few consistent findings of what variables influence knowledge in previous studies to inform predictions. We included all eight potential predictors (Age, Gender, Income, Education, UK Nation, Urbanicity, Dog History, Childhood Dog) in a global model. To identify the best models, we used an all-subsets approach, which can be appropriate for exploratory research when there is little a priori research [100,121]. Using the MuMin package in R (version 1.48.11) [122], we investigated 256 models for each item including the intercept and retained all models within 2 AICc units of the best-fitting models as candidates (see File S7 for candidate models). The ‘dredge’ function from the MuMin package also provided AICc weights for each model, which can be interpreted as the probability that a given candidate model is the best approximating one [100]. These are used to calculate the predictor weights, which estimate the relative importance of variables under consideration. This is calculated by summing the model AICc weights for the models in which each predictor is included, creating a ranking system for each predictor. If a predictor is in most models with the highest AICc weights, then its predictor weight will tend towards 1. Conversely, a predictor weight of 0 indicates that the predictor only appeared in models with no AICc weight, making it unlikely to be retained in the final model [100].

We provisionally retained predictors with predictor weights greater than 0.5 because this indicated that the predictor appeared in models accounting for at least 50% of the total AICc weight across the model set. We then compared a series of alternative models. These alternative models included a version of Dog History where the reference category was Current Owners, which combined First-Time Owners and Longtime Owners, and variables with predictor weights close to 0.5. We selected the model with the lowest AICc value and largest AICc weight as the final model. Some predictors were retained in the final model, but they were not significant at *p* < 0.05. In these cases, we report only significant results in Section 3 due to the volume of results. But all results, including non-significant ones and model selection results, are included in File S7.

Model diagnostics and other assumptions were checked. We tested the predictors’ potential multicollinearity by calculating an adjusted Variance Inflation Factor (GVIF) [119]. All results were approximately 1 and so no multicollinearity was present. We generated Pearson residuals and compared these to the fitted values [113]. Results also indicate no model misspecification. See File S7 for all model diagnostic results.

To provide an indication of model fit, we calculated McFadden’s Pseudo R^2^ and Nagelkerke R^2^ for all final models [123,124]. As these measures are sensitive to sample size, outcome distribution, and model specification [125], they are not directly comparable across models. Therefore, we calculated these values for each model to provide an indication of the relative improvement of a given model over its own null model (intercept-only model). These measures do not equate to R^2^ used in linear regression, but can provide an indication of substantive significance of the model [119]. There is currently limited guidance on benchmark values, especially for multinomial models [125]. However, McFadden’s Pseudo R^2^ is often lower than traditional R^2^, with McFadden [126] describing values of 0.2–0.4 as an excellent fit for logistic regression. Nagelkerke R^2^ values can range between 0 and 1 [124]. McFadden’s R^2^ and Likelihood Ratio Tests signalled that all models performed significantly better than their null models; however, these values varied between 0.01 and 0.30. Nagelkerke R^2^ ranged between 0.02 and 0.51. See File S7 for all model fit results.

## 3. Results

### 3.1. Demographic Characteristics of Participant Sample

The sample was approximately evenly distributed across the four UK nations, with each nation’s subsample nationally representative by age and gender according to Cint’s UK population benchmarks (see File S8 for target quotas and participant data). Urbanicity ranged between 0% (postcode district was completely rural) and 100% (completely urban). However, Urbanicity was skewed towards higher percentages of urban areas. The mean value was 77% and the median was 89%. The majority of the sample had some experience with dogs. Only 20% (358) reported having no lifetime experience with dogs, including not having a dog in childhood or living with a dog without owning one. See Table 3 for a full breakdown of the demographic characteristics of participants.

### 3.2. Distribution of Correct (Accurate), Uncertain (Don’t Know), and Incorrect Responses

Participants’ degree of accuracy and certainty varied widely across all items (see Table 4). Accuracy ranged from just 5% (82) of participants correctly identifying that Aversive Training Ban was not currently in force in the UK to 87% (1521) and 86% (1513) of participants correctly identifying that Restricted Breeds and Fouling Fines were currently in force across the UK. Although there was also considerable variation within each law grouping (current, nation-specific, and hypothetical laws), current laws were answered most accurately on average with a mean score of 66% compared to 34% for nation-specific laws and 38% for hypothetical laws.

Participants were most likely to answer incorrectly when responding to hypothetical laws that are not in force across the UK. More than half of participants incorrectly responded to 75% (3) of the nation-specific laws and 40% (4) of the hypothetical laws were currently in force in their respective nations. In contrast, incorrect responses were less likely on average for current laws. Only three of the eight current laws, Fear In Home, Lawful Shooting, and Collar ID, were answered incorrectly by more than 20% of participants. Instead, when participants answered current laws incorrectly, they were more likely on average to select ‘Don’t Know’ compared to nation-specific and hypothetical laws.

### 3.3. Demographic Variables Associated with Knowledge of Law

Dog History, Age, Gender, and Country were the most frequently retained variables after model selection. Dog History was included in the best supported models for all 22 law items, Age was included in the models for 16 law items, while Gender and UK Nation were included in the models for 11 law items (see Table 5). Income and Urbanicity were the only variables that showed some differences in their retention between current and hypothetical laws. Income was selected in just one current law model (Lawful Shooting) compared to five hypothetical law items. Urbanicity was only retained in current and nation-specific law items.

There was little consistency in the number of variables retained in the 22 best-supported models. Models for Fear In Home and Import Mutilation Ban both retained the least number of variables, keeping only Age and Dog History. Licencing and Dogs in Rentals models retained the most predictors of all items. Licencing retained all predictors except for Income, while Dogs in Rentals retained all but Urbanicity.

### 3.4. Results from Exploratory Multinomial Models

#### 3.4.1. Gender

Across all items, men tended to have greater odds of being incorrect or selecting ‘Don’t Know’ compared to women (see Table 6). Gender was significantly associated with knowledge for 45% (10) of the 22 items, four of which asked about current laws, five were associated with hypothetical laws, and one was associated with the nation-specific law Licencing. Men were always at greater odds of either selecting ‘Don’t Know’ or answering incorrectly for current laws. They were only both more uncertain and incorrect when answering Ear Cropping Ban with 51% increased odds of selecting ‘Don’t Know’ and 90% increased odds of answering incorrectly.

Men were at greater odds of being both more uncertain (selecting ‘Don’t Know’) and being more incorrect compared to women for all hypothetical laws except for Brachycephalic Breeding Ban and Prong Collar Ban. Restricted Neutering had the greatest difference, with men being at 222% (AOR: 2.22) greater odds of selecting ‘Don’t Know’ and 85.3% greater odds of being incorrect compared to women. The remaining AORs ranged between 27% and 53% increased odds of men selecting ‘Don’t Know’ and 32% to 85% increased odds of being incorrect as compared to women. Men were only more likely to be correct when answering Prong Collar Ban, where they had 40% reduced odds of being incorrect but were not significantly at reduced odds of selecting ‘Don’t Know’.

#### 3.4.2. Age

Overall, older participants tended to be more certain and answer with greater accuracy compared to younger participants. There were only three hypothetical laws (Import Mutilation Ban, Prong Collar Ban, and Import Mutilation Ban) and one nation-specific law (Leads Around Livestock) where younger participants answered with greater accuracy compared to older participants.

Age was significantly associated with knowledge for 68% (15) of the items overall (see Table 7). The only items where age was not selected in the best-supported models were Abandonment Ban, Straying Ban, and Collar ID in the case of the current law items, Shock Collar Ban for nation-specific laws, and Brachycephalic Breeding Ban and Restricted Euthanasia for hypothetical law items. Age effects had a high degree of confidence in estimates (smaller CIs and smaller *p*-values). Across all law items, Age had a mostly linear effect, and when non-linear, there was also a significant linear effect along with a quadratic or cubic effect. Welfare Transfer was the only exception to this, where there was only a significant 5th-level effect for answering incorrectly. This is likely a reflection of random noise, rather than a true effect [119].

For current laws, the results followed a very consistent pattern. As age increased, participants were more likely to answer accurately. Older participants were at reduced odds of answering incorrectly (AOR % ranged between −39% and −86%) and selecting ‘Don’t Know’ (AOR % ranged between −49% and −94%). The only exception to this pattern was Ear Cropping Ban. There were only significant non-linear differences between age groups when selecting ‘Don’t Know’, with no differences in answering incorrectly. Middle-aged categories answered with the least uncertainty, but there was an overall effect where the oldest participants answered with less uncertainty than the youngest participants.

Nation-specific laws had the most varied responses. When responding to Leads Around Livestock, older participants were more confident but also answered more incorrectly. They had 70% greater odds of answering incorrectly but had 55% reduced odds of selecting ‘Don’t Know’ compared to younger participants. When answering Licencing, older participants had 72% reduced odds of selecting ‘Don’t Know’ compared to younger participants, while the middle categories were the least likely to answer incorrectly compared to the youngest and oldest categories.

Age was selected for 80% (8) of hypothetical law items, with older participants answering with greater certainty and accuracy than younger participants for five of these laws. As age increased, participants had reduced odds of selecting ‘Don’t Know’ (AOR % ranged between −51% and −60%) and reduced odds of answering incorrectly (AOR % ranged between −59% and −83%). There were three exceptions to this pattern. Older participants were more likely to answer Prong Collar Ban, Import Mutilation Ban, and Aversive Training Ban incorrectly (AOR ranged between 2.83 and 3.94).

#### 3.4.3. Income

When significant effects were found, Income largely had a non-linear effect on knowledge. Income was only significantly associated with knowledge for 27% (6) of the 22 models (see Table 8). Only one of these were for the current law, Lawful Shooting, and Income was not significantly associated with knowledge for any nation-specific laws. The remaining five items were all hypothetical laws.

As income increased, participants tended to answer with greater certainty. All items had a negative linear association with knowledge when participants selected ‘Don’t Know’. Mandatory Exercise and Brachycephalic Breeding Ban only had linear effects, meaning that those with higher incomes were at reduced odds of selecting ‘Don’t Know’. Lawful Shooting, Public Access, and Left Alone Limit all had additional non-linear effects.

All significant results for answering incorrectly were positive quadratic effects, meaning that lower and higher incomes were at greater odds of answering incorrectly compared to middle incomes. Lawful Shooting was the exception to this, where middle incomes had higher odds of answering incorrectly compared to the lowest and highest incomes.

#### 3.4.4. Education

In a similar fashion to income, when significant effects were found, Education mostly had a non-linear, u-shaped curved effect on knowledge, particularly for hypothetical laws. Education was significantly associated with knowledge in 25% (2) of the eight current law models and 50% (5) of the ten hypothetical law models (See Table 9). However, the current law Abandonment Ban and the hypothetical law Brachycephalic Breeding Ban had only a non-linear fourth-level significant effect, which is likely capturing noise rather than a meaningful effect [119]. Education was significantly associated with only one nation-specific law, Licencing.

All significant results for Education were non-linear except for when participants selected ‘Don’t Know’ for Mandatory Exercise. As educational attainment increased, the odds of selecting ‘Don’t Know’ decreased by 45%. Additionally, Licencing and Left Alone Limit had linear effects with additional fourth-level effects when participants answered incorrectly. As educational attainment increased, participants had 93% higher odds of answering Licencing incorrectly, with a smaller fourth-level effect (AOR %: −30%) indicating a small deviation. In contrast, those with higher educational attainment had 53% reduced odds of answering Left Alone Limit incorrectly, with a larger fourth-level effect (AOR: 2.08) indicating that there is a more substantial deviation from the linear effect.

The other significant non-linear results, Ear Cropping Ban (current law), Licencing (nation-specific law), and Mandatory Exercise, Restricted Neutering, and Dogs In Rentals (hypothetical laws) had at least a positive quadratic effect. This means that those with the lowest and highest educational attainment had the highest odds of selecting ‘Don’t Know’ or answering incorrectly compared to middle categories. See File S7 for all results.

### 3.5. Residence

#### 3.5.1. UK Nation

Northern Irish participants answered with the greatest accuracy across the nations, while Welsh and English participants were the least accurate (see Table 10). Knowledge differed between UK Nations for 50% (11) of all 22 items. For the non-nation-specific laws, Northern Irish and Scottish participants differed most often from English participants. UK Nation was retained in 38% (3) of the eight current laws. When asked about Lawful Shooting, Northern Irish participants had 32% reduced odds of answering incorrectly, while Scottish participants had 33% reduced odds of selecting ‘Don’t Know’. Northern Irish participants were also at 45% reduced odds of selecting ‘Don’t Know’ for Fouling Fines but this effect was trending towards significance with a *p*-value of 0.053. The only instance where participants were more incorrect was Scottish participants when answering Collar ID. They had 64% increased odds of answering this item more incorrectly compared to English participants.

When answering hypothetical law items, Northern Irish participants answered with the greatest accuracy. Northern Irish participants had reduced odds of answering Dogs In Rentals (AOR: −50%) and Restricted Neutering (AOR: −63%) incorrectly compared to English participants. Scotland was also at reduced odds of answering Restricted Neutering incorrectly compared to English participants but to a lesser degree than Northern Irish participants (AOR: −38%). Scottish participants were more uncertain when answering Mandatory Exercise compared to English participants (AOR: 48%). Welsh participants only differed from English participants when answering Prong Collar Ban. They had 71% increased odds of selecting ‘Don’t Know’ compared to English participants.

UK Nation was significantly associated with legal knowledge for all four nation-specific laws; Lead Around Livestock and Licencing (in force in Northern Ireland), Shock Collar Ban (Wales), and Welfare Transfer (Scotland). Participants living in the nation where the law was currently in force always answered significantly more accurately. However, this is largely a result of participants across all nations responding to the item in a similar way, indicating that this is likely not simply a result of nation-specific knowledge (see Table 11). Instead, this may reflect an assumption across multiple UK nations that the law was in force even among nations where this is not the case. For example, 74% to 79% of participants from the four UK nations responded to Welfare Transfer as “True”, despite this only being currently in force in Scotland. This resulted in Scottish participants answering correctly, with the other participants from England, Northern Ireland, and Wales answering incorrectly, making them significantly different. Licencing was the only exception to this. While 75% (274) of Northern Irish participants correctly responded True, only 27% (122) to 38% (189) of participants from the other nations selected True, signalling a degree of nation-specific knowledge.

Based on these results, we conducted further exploratory analysis to test if participants from the UK Nations responded significantly differently to the nation-specific items. We ran an additional four multinomial models, one for each of the nation-specific laws, adding only UK Nation as the independent variable and their responses (True, False, Don’t Know) to the items as the dependent variable. We found significant differences between nations in how they responded to nation-specific laws (see Table 12). Although a ban on shock collars is only in force in Wales, a similar majority of participants in England and Scotland as in Wales thought (incorrectly) that this law was true for their nation. Northern Irish participants were the most accurate compared to Scotland and England when answering Shock Collar Ban, which is only in force in Wales. They had 95% increased odds of correctly selecting False compared to English participants but had 80% increased odds of selecting ‘Don’t Know’. Despite this, while they were more accurate than English or Scottish participants, the majority of Northern Irish participants falsely responded as if shock collars were banned in Northern Ireland.

Scottish and Northern Irish participants significantly differed from English participants when answering Welfare Transfer, which is only in force in Scotland. Both had reduced odds of selecting False compared to English participants, where Scottish participants had 42% reduced odds and Northern Irish participants had 45% reduced odds. Therefore, Scottish participants were more likely to answer correctly while Northern Irish participants were more likely to answer incorrectly compared to English and Welsh participants. There were no significant differences between nations for Leads Around Livestock, while there were significant differences when answering Licencing, despite both laws being in force only in Northern Ireland. Northern Irish participants were more likely to answer Licencing correctly by having 87% reduced odds of choosing False and 59% reduced odds of choosing ‘Don’t Know’. Scottish participants had 78% and Welsh participants had 49% increased odds of correctly selecting False compared to English participants.

#### 3.5.2. Urbanicity

Urbanicity was rarely significantly associated with knowledge. It was only selected for 25% (2) of current laws, 50% (2) of nation-specific laws, and no hypothetical laws. When Urbanicity was retained for nation-specific laws, participants in more urban areas answered more incorrectly compared to those in more rural areas (see Table 13). As Urbanicity increased, participants had 56% increased odds of answering Licencing incorrectly and 73% increased odds of answering Shock Collar Ban incorrectly. When answering current laws, participants living in more urban areas were more likely to differ from more rural participants in their uncertainty. As Urbanicity increased, participants had 210% (AOR: 2.1) increased odds of selecting ‘Don’t Know’ when responding to Lawful Shooting, while they had 47% reduced odds of being uncertain when responding to Fouling Fines.

### 3.6. Dog Ownership Experiences

Never Owners (those who reported never owning a dog as an adult) answered with greater uncertainty across all 22 law items but were only sometimes more incorrect when answering nation-specific and hypothetical laws (see Table 14). Never Owners were significantly more likely to select ‘Don’t Know’ compared to current dog owners when answering current laws. This effect ranged between 72% (AOR: 1.72) when Never Owners answered Lawful Shooting and 281% (AOR: 2.81) when answering Collar ID.

When answering nation-specific laws, Never Owners were more likely to answer with uncertainty compared to current dog owners (AORs: 1.75–3.74) but could also answer more incorrectly. Never Owners were more likely to answer Licencing and Shock Collar Ban incorrectly. Never Owners were at 252% (AOR: 2.52) greater odds of answering Licencing incorrectly compared to Current Owners and were at 38% (AOR: 1.38) increased odds of answering Shock Collar Ban incorrectly.

There was a similar pattern found when Never Owners answered hypothetical laws. Never Owners were always more likely to be uncertain and only sometimes more likely to answer incorrectly. Never Owners were least uncertain when answering Public Access (AOR: 1.81) compared to Current Owners, while they were most uncertain when answering Restricted Neutering (AOR: 4.34) compared to First-Time Owners. There were only four of the ten hypothetical questions where Never Owners answered more incorrectly compared to current dog owners. These were Mandatory Exercise (AOR %: 38%), Brachycephalic Breeding Ban (AOR %: 39%), Left Alone Limit (AOR %: 47%), and Restricted Euthanasia (AOR %: 64%).

Past Owners (those who have previously owned a dog as an adult but do not currently) were also more likely to be more uncertain when responding compared to current dog owners (see Table 15). However, this occurred less often than for Never Owners and when it did, the effect sizes were lower. Past Owners were at greater odds of selecting ‘Don’t Know’ for half of the current laws. They were most uncertain about Restricted Breeds (AOR: 2.04) compared to Current Owners. Past Owners were also more likely to answer more accurately compared to Current Owners when answering Ear Cropping Ban (AOR %: −49%). This was the only instance when Past Owners did not answer more incorrectly; however, they were also at greater odds of answering with greater uncertainty (AOR %: 45%) for this question compared to Current Owners.

Past Owners were also more uncertain about 75% (3) of the four nation-specific laws. The effect ranged between 52% increased odds of selecting ‘Don’t Know’ for Leads Around Livestock to having 208% (2.08) increased odds of answering Licencing with uncertainty compared to Current Owners. Past Owners were also more likely to answer Licencing (AOR %: 210%) and Shock Collar Ban (AOR %: 44%) incorrectly compared to Current Owners. When answering hypothetical laws, Past Owners were only at greater odds of selecting ‘Don’t Know’ with no difference in answering incorrectly compared to current dog owners.

Longtime Owners rarely differed significantly from First-Time Owners. When they did, Longtime Owners were more likely to answer current laws incorrectly but answered hypothetical laws with greater accuracy compared to First-Time Owners (see Table 16). For current laws, Longtime Owners had 70% (AOR: 1.70) and 234% (AOR: 2.34) increased odds of answering Lawful Shooting and Fear In Home incorrectly. When answering hypothetical laws, Longtime Owners had 68% (AOR: 1.68) reduced odds of answering Restricted Neutering incorrectly, but no difference in uncertainty compared to First-Time Owners. When responding to Brachycephalic Breeding Ban, Longtime Owners answered more accurately and with more certainty compared to First-Time Owners. Longtime Owners had 49% (AOR: 0.51) reduced odds of selecting ‘Don’t Know’ and 61% (AOR: 0.69) reduced odds of being incorrect. However, as there were only 82 Longtime Owners, these results should be interpreted with caution.

#### Childhood Dog

Across all items, there was a similar pattern whereby those who had a childhood dog were more likely to answer correctly or be more certain in their responses (see Table 17). Having a childhood dog was most often significantly associated with knowledge for hypothetical laws. Childhood Dog was retained for 50% (5) of the items, and in four of these, those with a childhood dog answered more accurately and with increased certainty compared to those who did not have a childhood dog. Childhood Dog was retained for three of the eight current laws. Those with a childhood dog were at reduced odds of selecting ‘Don’t Know’ when responding to Fouling Fines (AOR %: −62%), Straying Ban (AOR %: −24%), and Ear Cropping Ban (AOR %: −32%). Childhood Dog was only significantly associated with the nation-specific law Licencing. Those who had a childhood dog had 38% reduced odds of selecting ‘Don’t Know’ compared to those who did not have a childhood dog.

## 4. Discussion

Our results found that accurate legal knowledge among the UK public is limited. This suggests that the knowledge component of the ex ante function of law, the ability of law to shape future behaviour [22], may be limiting the law’s efficacy. Only two laws met the 80% threshold for adequate legal knowledge used by both Weng et al. [38] and Keogh et al. [36]; Restricted Breeds and Fouling Fines. In addition, at least half of the participants responded incorrectly to seven of the 14 laws that were not currently in force in their nation to be true. Taken together, these findings suggest not only that the UK population has insufficient knowledge of its legal responsibilities relating to dogs, consistent with prior research on legal knowledge in general [32] and dog-related laws [35,36,37,38], but also that members of the public may overestimate the extent to which the law protects dogs and their owners. In a context where budgets are constrained and enforcement of laws is inconsistent [25,110,127], the ability of law to shape behaviour before rules are broken becomes increasingly important. As knowledge is a component of this function, poor knowledge may become a barrier to the ex ante function of law operating effectively.

In this study, we interpreted both current and hypothetical laws as conceptually similar. As previous studies have found that people tend to have poor legal knowledge across different areas of law [32], including dog-related law [35,36,37,38], we expected legal knowledge to be shaped by assumptions and normative expectations for current, nation-specific, and hypothetical laws. Participants could be incorrect in this study in two ways: (1) by responding as if no law exists when it does in their respective nation, and (2) responding as if law exists when it does not in their respective nation. Therefore, we interpreted both forms of error as a potential result of a mismatch between people’s normative expectations of law and what the law currently is. Either form of mismatch could have potential benefits or have negative impacts for dogs, owners, or the public, depending on the nature of the law in question. For instance, the first type of error (assuming a law does not exist when it does), in some cases, has the potential to lead to better outcomes for dogs, such as incorrectly assuming that it is unlawful to shoot a dog if attacking livestock or to use a shock collar. However, we found that the second type of error, responding as if laws exist when they do not, was more common. This may suggest that as societal perception of dogs shifts towards more humanised and family roles, people may expect the law to be more protective of dogs and owners but may still have expectations for the way dogs should be managed in public spaces. While this interpretation is tentative, because in this study, most of our hypothetical laws asked about potential protections for dogs, future studies could broaden that focus to include laws that would hypothetically restrict dogs in the public and in the home.

These hypothetical and nation-specific laws produced a considerably higher degree of error compared to current law. A considerable proportion of participants incorrectly responded that they assumed laws not in force in their nation, or in the UK, to be true. At least half of the participants responded incorrectly to 40% (4) of the hypothetical laws and 75% (3) of the nation-specific laws as if these laws were in force in their specific nation. Participants’ responses to the nation-specific laws were especially illuminating. Participants across nations answered in similar ways to laws that were only in force in one UK nation. This indicates that responses were unlikely to be based on jurisdiction-specific legal knowledge. There are a number of possible non-mutually exclusive explanations. First, these results may be reflective of participants’ attitudes about what the law should be. This pattern would be consistent with prior research in criminal law, which found that individuals lacked accurate knowledge of the laws applicable in their own jurisdiction, and instead their responses reflected their normative views of the issues presented [13,128].

Second, it may be a result of participants’ exposure to UK-wide and global media. Ofcom, the regulatory authority for the UK’s media, found that the most common sources of news were a UK-wide news channel and Facebook, a global social media site [129,130,131]. Research indicates that people in the devolved nations may not be able to accurately identify when UK-wide information relates to their nation or to England, where UK-wide media often operates from. A report from Cardiff University and YouGov [132] found that Welsh participants could not accurately identify when information in examples of UK-wide news stories applied in Wales or England. Interestingly, Northern Ireland has been found to engage the most with nation-specific television and radio across the devolved nations [133]. This may be a potential reason why Northern Irish participants were the most accurate of the four nations, consistent with the PDSA’s Paw Report [134] which also found that Northern Irish participants had higher self-reported awareness of the five welfare needs in the Animal Welfare Act. As localised news and media organisations are declining in many countries across the world [135,136,137], future research could examine the role of country-wide media and the opinions of law, and how these can interact to influence localised legal knowledge.

### 4.1. Law Knowledge Varied Across Issues

We found that legal knowledge varied substantially across items. This aligns with Keogh et al. [36] who also found that Irish participants surveyed had different degrees of accuracy across different types of current law. While in our study, this variation was greater for hypothetical laws, participants tended to answer current laws more accurately on average. This suggests that some people do have a sense of what current laws are, despite studies consistently finding inadequate levels of knowledge across legal domains [32,35,37,38]. Instead, legal knowledge may not be universally poor and is issue dependent. This may be due to the increased complexity and volume of law making it impossible for people to know all of their legal responsibilities [30,32,68]. In contrast to previous studies [32,35,36], we found a variety of demographic variables to be significantly associated with knowledge. This suggests that legal knowledge is opportunistically acquired, obtained through exposure to media messages, life experiences, or inferred through intrinsic normative beliefs rather than obtained formally.

However, the difference in variation and average accuracy between current and hypothetical laws may be a result of the law’s beneficiaries. Most of our current laws govern the ‘public dog’, while most of the hypothetical dog laws govern the ‘private dog’. This was partly a result of there being few current ‘private dog’ laws in the UK that met our inclusion criteria of creating distinct boundaries of behaviour. Weir et al. [65] found that laws governing dogs in public prioritised the interests of the general public while deprioritising dogs and their owners. This may suggest that people acquire more knowledge of laws that prioritise the general public. Other countries have taken a different approach to dog welfare laws, creating more prescriptive legislation, particularly in Germany, Sweden, and the Netherlands [80]. Future studies could compare knowledge of laws governing the ‘public dog’ and ‘private dog’ in other countries where there are laws that create clearly defined boundaries of behaviour for both dog control and welfare laws, which may allow for more direct comparisons.

Laws that govern the ‘public dog’ and prioritise the general public may be more visible, resulting in greater public knowledge on average. Laws that are publicised more often and with more consistency may result in more people developing more accurate knowledge. A key aspect of the ex ante function of law operating effectively is that laws are adequately published so people can be made aware of their legal responsibilities [13]. We found that Restricted Breeds and Fouling Fines were the only laws for which over 80% of participants answered correctly. Both issues receive substantial media attention and are frequently highlighted in public discourse [37,138,139]. Dog fouling is a highly emotive issue, shaped by fears of disease, feelings of disgust, and it is a possible symbolic representation of civil decline and neglect [57,139,140]. As a result, non-compliance with dog fouling legislation is frequently reported to councils [139,140,141] and prompts research focused on finding the most effective messaging to encourage owners to pick up after their dog [142]. These efforts have contributed to the widespread use of signage and government campaigns [143,144]. It is likely that people noticing these signs contributed to the high accuracy found in this study. Williams et al. [72] found that 88% of participants reported observing signs displaying dog leashing laws, while Zamora-Nasca & Lambertucci [95] found that increased clarity of regulations managing dogs’ access to protected areas in Argentina was associated with participants’ improved knowledge of them. The frequency and visibility of signage may influence public knowledge of regulation, and future research could examine this relationship directly.

Media sources and public discourse may also inform legal knowledge. Restricted Breeds was accurately answered by 87% of participants. In contrast, we found relatively low levels of knowledge and high uncertainty for Fear In Home, despite being contained in the same Dangerous Dogs Act 1991 in England, Scotland, and Wales [145]. Media reports concerning dog attacks and bites often focus on the breed of dog, attributing attacks to specific breeds such as Staffordshire Terriers and Pit Bulls, depending on the time period [146,147]. Parkinson et al. [148] found that 97% of UK participants surveyed had read or seen a news story about a dog attack and the majority of these participants reported remembering the breed featured in the story. This emphasis on breed may have increased participants’ knowledge of laws relating to restrictions on banned breeds rather than owners’ responsibility to prevent their dogs from causing apprehension to others in both public and private spaces. Notably, breed-specific legislation was highly visible during data collection for the present study. Debates were ongoing over adding XL Bullies to the list of banned breeds, which may have contributed to increased knowledge of Restricted Breeds [149,150] compared to Fear In Home, which remained unchanged in the law.

#### 4.1.1. Dog Owners Answered with Greater Certainty but Not More Accurately

Experience of the issues presented may also play an important role. As dog-related law has moved to regulating owners rather than dogs directly [6,30], dog owners are more likely to encounter situations or messaging that make them aware of their legal requirements. We found that the experience of dog ownership was the most consistently important variable, being significantly associated with all 22 items. Those who had never owned a dog primarily drove these results by answering with more uncertainty for every item. While those who previously owned a dog were also more likely to be uncertain than current owners, they were uncertain for fewer items and to a lesser degree (they had smaller effect sizes) than those who had never owned a dog. Although having a childhood dog was rarely associated with knowledge, when significant, participants answered with more accuracy and confidence. Together, these results suggest that exposure to dogs provided participants with greater confidence when answering but not necessarily more accuracy.

This may be due to dog owners’ information sources. There is limited information about where dog owners get legal information from. In one of the few papers to address this, Oxley et al. [37] found that the internet and television were the most common sources of information for those who could name at least one banned breed, compared to just 7% of participants who cited government materials. However, the media environment has transformed since the early 2010s, with social media first becoming mainstream around this time [151]. Survey results from other areas of dog ownership find that websites, online forums, dog television shows, and friends and family are the most common sources of information, depending on the topic, which are unlikely to be location-specific [148,152,153,154]. However, some participants reported they found conflicting advice and did not know what information to trust [148,153], and academic research has found that online advice rarely has reliable indicators of quality [155]. As research in other non-animal-related academic fields has found that people tend to not have the appropriate skills to evaluate online information [156,157], dog owners may be unable to identify reliable information sources. Studies also find that dog owners often reference themselves or their own previous ownership experience as their main source of information [153,158], suggesting that dog owners are confident in their knowledge but likely acquire this through unreliable or low-quality means.

#### 4.1.2. Older Participants Often Answered with Greater Accuracy and Confidence

Instead, greater life experience that comes with age may provide people with more opportunities over time to encounter a wider range of experiences and messaging, which may lead to more accurate knowledge. We found that age was frequently associated with knowledge, with older participants more likely to answer with greater accuracy and confidence, particularly for current laws. However, when laws change, previously accumulated knowledge may result in confident but incorrect knowledge. Licencing was unique amongst the items, being the only law item that was previously a UK-wide law. It was abolished in England, Scotland, and Wales in 1987 due to widespread non-compliance but retained in Northern Ireland [24]. We found that as age increased, people’s certainty in their responses increased, but both the youngest and oldest participants were more likely to answer incorrectly compared to those in middle age groups. The youngest participants in England, Scotland, and Wales were born after dog licencing was abolished and so did not experience applying for licences and were not exposed to messaging about the requirement. The oldest participants likely would have been, particularly older dog owners. Therefore, while accumulated experience over time may allow people to develop greater breadth of knowledge, when laws change, this knowledge may not be updated.

However, experience alone is unlikely to explain all results. Younger participants were more likely to respond incorrectly or be more uncertain about hypothetical laws that provided more rights to owners (Public Access and Dogs In Rentals) or stricter prescriptive welfare requirements for dogs (Mandatory Exercise, Left Alone Limit, and Restricted Neutering). Older participants were more likely to incorrectly respond as if Prong Collar Ban, Aversive Training Ban, and Import Mutilation Ban were currently law. Previous research has found that older participants are more likely to use aversive training methods than younger participants [159], while younger people have been found to be less likely to declare pets to landlords when renting [160]. These examples suggest that these groups may have greater experience with the issues that they falsely assumed law provided more protections for.

These results suggest that factors other than increased experience of issues may be playing a role in the formation of legal knowledge. Changing perceptions of animal welfare and attitudes towards dogs may be contributing to these results. Animal welfare has developed considerably since the 1960s, increasingly focusing on promoting positive welfare states rather than solely focusing on preventing negative ones [21,161,162,163,164]. Therefore, the differences between younger and older participants’ knowledge may reflect their attitudes towards how the law should protect dog welfare. The gender differences we found also support this explanation. Men tended to respond with greater inaccuracy to items that are in line with their opinions found in prior studies, such as neutering [165], convenience euthanasia [166], straying [167], and the use of aversive training methods [168]. As non-animal legal studies have found that legal knowledge can align with people’s beliefs about what the law should be [32], future research should seek to disentangle legal knowledge from attitudes towards law to gain a greater understanding of the role attitudes play in animal-related legal knowledge.

### 4.2. Implications for Conflict, Institutional Trust, and Third-Party Reporting

Variation in accuracy and uncertainty across the law items, as well as demographic and geographic differences in legal knowledge, may have the potential to exacerbate conflict. Differing degrees of certainty of legal rules between groups may be as impactful as opposing perceptions of the law, as uncertainty may lead to the development of informal rules. Eldridge & Jović [17] describes dog owners’ frustrations over inconsistent access to public transport based on the knowledge and attitudes of the drivers and other passengers. Increased signs in public spaces or clear guidelines publicised in easy-to-access online sites, detailing what the rules are, may be an effective strategy to reduce uncertainty. Zamora-Nasca & Lambertucci [95] found that the accessibility and clarity of regulations influenced people’s knowledge and acceptance of rules concerning dogs’ access to protected parks in Argentina. In particular, they found that the participants surveyed were more likely to not know if dogs were allowed in protected parks in Argentina if there was no publicised information. Only 7% of our participants were uncertain when answering Fouling Fines, and this is the only law in our selection of items where there are lots of visible signs [143]. Further research is required to gain insight into whether the availability and clarity of rules can reduce people’s uncertainty or inaccurate knowledge about laws.

The high degrees of uncertainty, particularly when answering the hypothetical laws, may have the potential to erode public trust and institutional legitimacy. Particularly relevant is our finding that approximately three-quarters of Northern Irish, English, and Welsh participants incorrectly responded as if an inspector could transfer ownership if an animal is deemed to be suffering unnecessarily, rather than requiring a court decision. Kennelling dogs for long periods while cruelty cases are prosecuted has been described as a major barrier to intervening in such cases [23]. Our results suggest that participants from Northern Ireland, England, and Wales may not understand this barrier and may therefore struggle to interpret inaction following a report of animal cruelty. Evidence from other areas of law enforcement and regulations has found that unsuccessful reporting experiences can erode trust in the institutions responsible for providing protections and can discourage future reporting [169]. Therefore, if people report instances of what they perceive to be unlawful animal cruelty and subsequently learn that the action is not banned by law or no action is taken, they may lose institutional trust and be less likely to report other cases of suspected animal abuse. This confusion also has implications for those tasked with enforcing the law. In the United States, Moss et al. [26] found that enforcement officers found some laws difficult to enforce because they were unsure about where the boundary of rule breaking was. Further research could explore the experiences of those enforcing dog-related laws and how the clarity of rules and public knowledge of them impacts their ability to do their jobs effectively.

### 4.3. How to Improve Legal Knowledge

Frequently, education and awareness campaigns are discussed as a key method to improve knowledge of laws [12,29,76]. However, there are a number of barriers to successfully achieving this, such as campaigns lacking clearly defined goals [12] and the need for testing educational campaigns before widespread release [170]. We found that current dog owners were not generally more accurate when responding to questions but were more confident in their responses compared to non-owners, creating what Philpotts et al. [171] describes as the challenge of educating owners who do not always believe they have poor knowledge. Understanding what variables influence knowledge may help make awareness campaigns more targeted. For example, there are reports that ear cropping has become increasingly prevalent and popular in the UK [110]. However, we found that 72% of our sample accurately knew that this practice was banned, suggesting that most people are aware that this practice is outlawed. Therefore, widespread awareness campaigns may not be required. Instead, as we found that men and younger participants were most likely to answer incorrectly, dedicated education campaigns focused on these groups may be more successful.

Although knowledge of legal rules helps individuals recognise legal boundaries, increasing legal knowledge does not necessarily lead to compliance. Compliance is also shaped by social norms, capacity to comply or offend, the predictability of enforcement and severity of punishment for non-compliance, and individuals’ rational decisions to comply based on a cost–benefit analysis [33]. This suggests that while knowledge of law is important, improving it may not necessarily lead to an increase in compliance [32]. Studies of dog owners have found that even when they are aware of behaviours that can improve their dog’s welfare, they do not always engage in them [83,172]. We found that approximately three-quarters of Northern Irish participants accurately identified that all dogs legally require a licence despite reports of widespread non-compliance [173]. As studies consistently find legal knowledge to be inadequate, more research is needed into the effectiveness of other solutions such as environmental design, more consistent enforcement, and greater individual support for owners to ensure they are capable of meeting their legal obligations.

### 4.4. Future Directions and Limitations

This study has several notable strengths. To our knowledge, it is the first to use a nationally representative survey to assess people’s knowledge of dog law across the UK, with equal representation from all four nations and comparable numbers of dog owners and non-dog owners. This design allowed for meaningful comparison between groups, which enabled us to identify multiple variables that influenced knowledge.

One limitation was the unusually high proportion of first-time dog owners and those who did not have a childhood dog within the sample. While 90% of our current dog owners were classified as first-time owners, the PDSA [82] found that 42% of dog owners were first-time dog owners in the same year as data collection took place. This may have been a result of how a participant’s dog history was collected. We provided participants with a list of different options and directed them to select all that applied. These options included ‘currently own at least one dog’, ‘previously owned a dog as an adult’, ‘had a dog in the household as a child’ and ‘never owned a dog’. It is possible that participants who currently owned a dog selected the response ‘currently own at least one dog’, which was always listed first, and did not read the rest of the options. As we created groups based on these responses (e.g., Longtime Owners were classified as those who selected that they currently own a dog and also selected that they had previously owned a dog), this may have reduced the number of Longtime Owners and those with a childhood dog. To ensure participants were attentive for the remaining questions, we conducted data quality checks and found no evidence of straight-lining or randomly selected responses (see File S8).

This may have resulted in more conservative comparisons, making it more difficult to detect statistically significant effects due to the smaller-than-expected sample sizes of Longtime Owners and those who had dogs during childhood. When differences were significant, Longtime Owners were at greater odds of answering Lawful Shooting and Fear In Home incorrectly while answering Restricted Neutering and Brachycephalic Breeding Ban correctly, while those with childhood dogs were more likely to answer correctly and definitively. Given the diversity of dog owners [39,46,174], future research would benefit from recruiting larger samples of dog owners with different experiences with dogs, including the choice of dog breed, strength of human–dog relationship, and pet parenting style.

Several findings also highlight productive avenues for further investigation. Urbanicity played a limited role in the present analyses, likely reflecting the predominantly urban distribution of the UK population and variation in how rurality is classified across nations [104,105,106]. However, when Urbanicity was significant, results suggest important differences that may reflect the priorities of rural and urban communities. Urban participants were more likely to answer Shock Collar Ban incorrectly and Lawful Shooting with greater uncertainty compared to rural participants. Both items are aimed at stopping livestock attacks, an issue of particular relevance in rural communities [175]. Electronic collars (also known as e-collars and shock collars) have been linked to the prevention of livestock attacks by their proponents, the media, and some farming bodies, particularly in Wales, where they have been banned [176]. Interestingly, there was no significant difference between rural and urban participants when answering Leads Around Livestock, a law aimed at preventing livestock attacks without causing direct harm to dogs. Future studies could adopt more consistent or targeted measures of rural and urban experience to better capture these dynamics.

Future studies could further investigate why knowledge varies across different issues, a finding that aligns with Keogh et al. [36]. This study provides useful background for future studies that focus on the degree to which legal knowledge, or incorrect assumptions of the law, may shape compliance behaviours, reported experience with enforcement mechanisms, reporting behaviour, institutional trust, and societal conflict. This may help create more effective interventions to improve legal knowledge and compliance. Similarly, future research could also explore the role of legal consciousness, which describes how legal knowledge can only be ‘activated’ when the situation is encountered [177]. Scenario-based or vignette-driven study designs could therefore complement standard survey approaches to better assess how legal knowledge operates in practice.

It is worth acknowledging that a high level of legal knowledge or compliance does not necessarily imply that optimal outcomes for society or for specific stakeholders will be accrued as a result. Indeed, an intended function of law is to balance the interests of different groups within society and reduce social conflicts [65]; hence, even when working as intended, law may provide protection or benefits for one party, such as the public, over another, such as dogs. Furthermore, it is plausible that even if all laws in force at a given moment were fully complied with, they would not necessarily deliver the intended benefits nor prevent intended or unintended harm from occurring. This is because these outcomes are additionally a function of the nature of laws themselves. For example, almost all participants accurately responded to Restricted Breeds, which is part of legislation that has been widely criticised for being poorly drafted, using an ineffective approach to reduce dog attacks, and for causing harm to dogs [68,138,178]. Therefore, compliance with this law may not necessarily result in ‘public good’ and may cause harm, including to dogs, in the process. Judgement over the realised benefits or harms of specific laws is beyond the scope of the evidence we consider within the present study. Future studies could focus on the experiences of dog owners subject to laws to gain a deeper understanding of whether public knowledge is reflective of contested law, weak legitimacy, or misalignment between legal design and real-world conditions.

Finally, while this study focused on dogs due to their distinctive legal and social status, these results have broader relevance for other companion animals. Cats, in particular, are facing greater legal scrutiny and face many of the same welfare issues as dogs [110,179,180], as well as undergoing a shift in societal perception towards becoming incorporated into more-than-human families [181]. As other companion animals are increasingly legislated, future studies to assess owners’ legal knowledge may be required.

## 5. Conclusions

This study provides evidence that people’s knowledge of dog law in the UK is generally limited but unevenly distributed across different topics of legislation, particularly across current, nation-specific, and hypothetical laws. Dog ownership history and age played a prominent role in participants’ confidence and accuracy when responding to laws. Participants, particularly younger participants, were especially error-prone when responding to hypothetical and nation-specific laws that provided dogs and owners with greater legal protection. This suggests that legal knowledge is unlikely to be uniformly guiding behaviour, and instead, there may be gaps between the law in practice and people’s perceptions of it. In a context of limited enforcement and individualised responsibility placed on dog owners, who were more confident but not more accurate in their responses, these results have potential consequences for conflict, third-party reporting, and institutional trust and legitimacy. Together, these findings suggest that legal knowledge is unlikely to be playing an effective role in the ex ante function of law and that people may imagine a more protective and developed legislative environment for dogs than currently exists in the UK.

## Figures and Tables

**Table 1:** Dog History group definitions.

Dog History Group	Definition	Sample Size
Current Owners	All participants who selected that they currently own at least one dog. Combines First-Time Owners and Longtime Owners.	831
First-Time Owners	Those who selected currently own at least one dog but did not select previously owned a dog as an adult.	749
Longtime Owners	Participants who selected currently own at least one dog and selected previously owned a dog as an adult that is not their current dog(s).	82
Past Owners	Participants who are not current owners but selected previously have owned at least one dog as an adult in the past.	333
Never Owners	Participants who do not currently own a dog and have never owned a dog as an adult.	594

**Table 2:** Items presented to participants, with the sources used to identify them and their legal status (country in which they are in force) at the time of the survey design. The short names are reflective of the language used in the items’ phrasing. The nation or country where the nation-specific and hypothetical laws are based on are included in brackets. NI is shorthand for Northern Ireland.

Statement	Short Name	Legal Status	Source
It is unlawful for an owner to abandon their dog.	Abandonment Ban	Current	[36]
Certain breeds must be muzzled and on a leash in a public place.	Restricted Breeds	Current	[36]
It is illegal for a dog to cause fear or apprehension in another person in your home or someone else’s home.	Fear In Home	Current	[65]
Owners can be fined if their dog fouls in a public place.	Fouling Fines	Current	[36]
Dogs must at all times wear a collar that bears the name and address of the owner inscribed on a plate, badge, or disc.	Collar ID	Current	[36]
It is legal for someone to shoot a dog if they are worrying/attacking livestock.	Lawful Shooting	Current	[65]
It is unlawful for an owner to allow their dog to stray.	Straying Ban	Current	[36]
It is illegal to crop a dog’s ears.	Ear Cropping Ban	Current	[65]
It is unlawful for a dog to be off lead when around livestock.	Leads Around Livestock	Nation-specific (NI)	[65]
All dog owners must have a licence for their dog(s).	Licencing	Nation-specific (NI)	[36]
Any dog who is found to be suffering unnecessarily can be removed by a government-authorised person and transferred to another owner without the current owner’s consent.	Welfare Transfer	Nation-specific (Scotland)	[65]
It is unlawful to use a collar that gives an electric shock to a dog.	Shock Collar Ban	Nation-specific (Wales)	[65]
Dogs should be walked at least every 6 h by law during the day unless there are welfare reasons.	Mandatory Exercise	Hypothetical (Sweden)	[80]
All dogs have the legal right to enter restaurants, bars, and shops with their owners as long as the dog is not a threat to public safety.	Public Access	Hypothetical (Spain)	[107]
Dogs can only be euthanised if there is a reasonable reason to do so.	Restricted Euthanasia	Hypothetical (Austria)	[80]
It is illegal to breed flat-faced dogs like Pugs and French Bulldogs.	Brachycephalic Breeding Ban	Hypothetical (Netherlands)	[80]
It is unlawful to import a dog with cropped ears or a docked tail.	Import Mutilation Ban	Hypothetical (UK Bill)	UK stakeholders
A dog cannot be left alone for more than 6 h by law.	Left Alone Limit	Hypothetical (Sweden)	[80]
It is unlawful to neuter or spay a dog unless for a health reason.	Restricted Neutering	Hypothetical (Norway)	[80]
It is illegal to use a spiked collar (also known as a prong collar).	Prong Collar Ban	Hypothetical (Sweden)	[80]
All owners can legally keep a dog in their rented accommodation.	Dogs In Rentals	Hypothetical (France)	UK stakeholders
It is illegal to train animals in a way that inflicts significant pain, distress, or harm to the animal.	Aversive Training Ban	Hypothetical (Germany)	[80]

**Table 3:** Demographic characteristics of participants (*n* = 1758). ‘Missing’ represents participants whose postcode districts did not match their nation of residence and so were set to missing. ‘Prefer not to say’ responses were also set to missing.

Variable	Levels	Number	Percentage of Variable Total
Age	18–24	200	11%
	25–34	302	17%
	35–44	288	16%
	45–54	310	18%
	55–64	287	16%
	65+	371	21%
Gender	Female	883	50%
	Male	869	49%
	Non-binary	1	0%
	Other gender identity	3	0%
	Prefer not to say	2	0%
Education	High school/secondary school or equivalent	700	40%
	Apprenticeship/Trade school	155	9%
	First-degree-level qualification	541	31%
	University higher degree (e.g., MSc, PhD)	310	18%
	None of the above	43	2%
	Prefer not to say	9	1%
Income	Less than £10,000	124	7%
	£10,001–£20,000	255	15%
	£20,001–£30,000	349	20%
	£30,001–£40,000	304	17%
	£40,001–£60,000	348	20%
	£60,001–£80,000	167	10%
	£80,001–£100,000	81	5%
	Over £100,000	63	4%
	Prefer not to say	67	4%
UK Nation	England	495	28%
	Northern Ireland	360	21%
	Scotland	446	25%
	Wales	445	25%
	Missing	12	1%
Dog History	First-time dog owner (First-Time Owners)	749	43%
	Longtime current dog owner (Longtime Owners)	82	5%
	Never owned a dog (Never Owners)	594	34%
	Previous dog owner but not current (Past Owners)	333	19%
Childhood Dog	No	1292	74%
	Yes	466	27%

**Table 4:** Participants’ accuracy and certainty varied across law items. Law items are split into current, nation-specific, and hypothetical laws and ranked by participant accuracy (percentage of participants who responded correctly as to whether the law item was true or false), with the number of participants included in brackets. For nation-specific laws, brackets denote where the law is in force. NI signifies that the law is only in force in Northern Ireland, S in Scotland only, and W in Wales only. See Table 2 for definitions of the items’ short names.

Law Status	Items	Correct	Don’t Know	Incorrect
Current Laws	Restricted Breeds	87% (1521)	9% (152)	5% (85)
	Fouling Fines	86% (1513)	7% (130)	7% (115)
	Abandonment Ban	77% (1359)	14% (239)	9% (160)
	Ear Cropping Ban	72% (1265)	20% (356)	8% (137)
	Straying Ban	59% (1034)	24% (427)	17% (297)
	Collar ID	52% (912)	19% (338)	29% (508)
	Lawful Shooting	51% (899)	26% (454)	23% (405)
	Fear In Home	47% (829)	29% (508)	24% (421)
	Mean (Median) ± SD	66% (65%) ± 16%	19% (20%) ± 8%	15% (13%) ± 9%
Nation-Specific Laws	Licencing (NI)	59% (1038)	13% (220)	28% (490)
	Shock Collar Ban (W)	29% (517)	18% (309)	52% (922)
	Leads Around Livestock (NI)	25% (442)	20% (346)	55% (960)
	Welfare Transfer (S)	24% (426)	18% (316)	57% (1007)
	Mean (Median) ± SD	34% (27%) ± 17%	17% (18%) ± 3%	48% (54%) ± 14%
Hypothetical Laws	Public Access	71% (1247)	16% (284)	13% (227)
	Restricted Neutering	69% (1211)	16% (290)	15% (257)
	Mandatory Exercise	57% (1003)	25% (445)	18% (310)
	Dogs In Rentals	57% (997)	21% (370)	22% (391)
	Brachycephalic Breeding Ban	47% (827)	32% (559)	21% (372)
	Left Alone Limit	37% (642)	37% (644)	27% (472)
	Restricted Euthanasia	16% (274)	23% (399)	62% (1085)
	Import Mutilation Ban	14% (251)	35% (617)	51% (890)
	Prong Collar Ban	12% (207)	26% (455)	62% (1096)
	Aversive Training Ban	5% (82)	10% (182)	85% (1494)
	Mean (Median) ± SD	38% (42%) ± 25%	24% (24%) ± 9%	38% (25%) ± 25%

**Table 5:** Dog History and Age were the predictors most often significantly associated with law knowledge in the best-supported multinomial models. Cells are highlighted when the variable was selected to be included in the model and was significant at *p* < 0.05. The reported McFadden’s R^2^ was calculated using the NA dataset (all missing data set to NA values), but all results can be found in File S7.

Sphere	Short Name	Gender	Age	Education	Income	UK Nation	Urbanicity	Dog History	Childhood Dog	McFadden’s R^2^
Current Laws	Restricted Breeds									0.10
	Fouling Fines									0.05
	Abandonment Ban									0.02
	Ear Cropping Ban									0.06
	Straying Ban									0.01
	Collar ID									0.02
	Lawful Shooting									0.03
	Fear In Home									0.02
Nation-Specific Laws	Licencing									0.09
	Shock Collar Ban									0.20
	Leads Around Livestock									0.11
	Welfare Transfer									0.30
Hypothetical Laws	Public Access									0.07
	Restricted Neutering									0.11
	Mandatory Exercise									0.05
	Dogs In Rentals									0.06
	Brachycephalic Breeding Ban									0.03
	Left Alone Limit									0.05
	Restricted Euthanasia									0.02
	Import Mutilation Ban									0.02
	Prong Collar Ban									0.04
	Aversive Training Ban									0.03
% (*n*) of Current Laws Significant		50% (4)	50% (4)	25% (2)	13% (1)	38% (3)	25% (2)	100% (8)	38% (3)	
% (*n*) of Nation-Specific Laws Significant		25% (1)	75% (3)	25% (1)	0% (0)	100% (4)	50% (2)	100% (4)	25% (1)	
% (*n*) of Hypothetical Laws Significant		50% (5)	80% (8)	50% (5)	50% (5)	40% (4)	0% (0)	100% (10)	50% (5)	
% (*n*) of Total Variable Laws Significant		45% (10)	68% (15)	36% (8)	27% (6)	50% (11)	18% (4)	100% (22)	41% (9)	

**Table 6:** Significant results from multinomial models showed that men generally answered more incorrectly and with more uncertainty than women. Correct was the baseline and so it is not shown in the table. Adjusted odds ratios (AORs) are calculated by exponentiating the Estimate. SE is the Standard Error, the LL is the lower limit, and UL is the upper limit of the 95% Confidence Interval (CI). Bolding signifies significant results at *p* < 0.05. The item name is bolded when both Incorrect and Don’t Know responses are significantly influenced by Gender. Incorrect responses are italicised to aid comparison. Adjusted odds ratios (AORs) greater than one indicate increased odds of the response by men as compared to women, while those AORs of less than one indicate reduced odds. Models only compared men versus women after removing other gender identities due to low frequencies. Please see File S7 for all non-significant results.

Status	Item	Response	Estimate	SE	AOR	95% CI		*p*
						*LL*	*UL*	
Current Laws	Restricted Breeds	**Don’t Know**	**0.77**	**0.18**	**2.16**	**1.51**	**3.10**	**<0.001**
		*Incorrect*	*0.11*	*0.23*	*1.11*	*0.71*	*1.75*	*0.64*
	**Ear Cropping Ban**	**Don’t Know**	**0.41**	**0.13**	**1.51**	**1.18**	**1.94**	**0.001**
		***Incorrect***	***0.64***	***0.19***	***1.90***	***1.31***	***2.76***	***<0.001***
	Straying Ban	Don’t Know	−0.01	0.12	0.99	0.78	1.24	0.900
		***Incorrect***	***0.40***	***0.13***	***1.49***	***1.15***	***1.94***	***0.003***
	Collar ID	Don’t Know	0.08	0.13	1.09	0.84	1.40	0.519
		***Incorrect***	***0.36***	***0.11***	***1.43***	***1.15***	***1.78***	***0.002***
Nation-Specific Laws	Licencing	Don’t Know	0.24	0.16	1.27	0.93	1.74	0.134
		***Incorrect***	***0.26***	***0.12***	***1.30***	***1.03***	***1.63***	***0.028***
	Leads Around Livestock	Don’t Know	0.10	0.15	1.10	0.82	1.48	0.523
		*Incorrect*	*−0.20*	*0.13*	*0.82*	*0.64*	*1.05*	*0.122*
Hypothetical Laws	**Restricted Neutering**	**Don’t Know**	**0.80**	**0.15**	**2.22**	**1.67**	**2.95**	**<0.001**
		***Incorrect***	***0.62***	***0.15***	***1.85***	***1.38***	***2.48***	***<0.001***
	**Dogs In Rentals**	**Don’t Know**	**0.28**	**0.13**	**1.32**	**1.02**	**1.71**	**0.035**
		***Incorrect***	***0.58***	***0.13***	***1.79***	***1.38***	***2.32***	***<0.001***
	Brachycephalic Breeding Ban	**Don’t Know**	**0.24**	**0.12**	**1.27**	**1.01**	**1.60**	**0.038**
		*Incorrect*	*0.18*	*0.13*	*1.19*	*0.92*	*1.55*	*0.178*
	**Restricted Euthanasia**	**Don’t Know**	**0.43**	**0.16**	**1.54**	**1.12**	**2.10**	**0.007**
		***Incorrect***	***0.28***	***0.14***	***1.32***	***1.01***	***1.72***	***0.044***
	Prong Collar Ban	Don’t Know	−0.11	0.17	0.90	0.64	1.26	0.539
		***Incorrect***	***−0.52***	***0.16***	***0.60***	***0.44***	***0.81***	***0.001***

**Table 7:** Significant results from multinomial models showed that accuracy and certainty mostly increased with Age. Correct was the baseline and so it is not shown in the table. Adjusted odds ratios (AORs) are calculated by exponentiating the Estimate. SE is the Standard Error, LL is the lower limit, and UL is the upper limit of the 95% Confidence Interval (CI). Age was modelled as an ordered factor using polynomial contrasts, and only significant contrasts are reported. For linear effects, adjusted odds ratios (AORs) greater than one indicate that as age increases, the odds of the responses increase, while AORs less than one indicate decreased odds. Please see File S7 for all results for each polynomial orthogonal contrast including non-significant results. Incorrect responses are italicised to aid comparison.

Status	Item	Response	Contrast	Estimate	SE	AOR	95% CI		*p*
							*LL*	*UL*	
Current Laws	Restricted Breeds	Don’t Know	Linear	−1.96	0.25	0.14	0.09	0.23	<0.001
		*Incorrect*	*Linear*	*−2.86*	*0.49*	*0.06*	*0.02*	*0.15*	*<0.001*
	Fouling Fines	Don’t Know	Linear	−1.17	0.24	0.31	0.19	0.50	<0.001
		*Incorrect*	*Linear*	*−1.00*	*0.26*	*0.37*	*0.22*	*0.61*	*<0.001*
	Ear Cropping Ban	*Incorrect*	*Linear*	*−0.98*	*0.24*	*0.37*	*0.23*	*0.60*	*<0.001*
		*Incorrect*	*Quadratic*	*0.56*	*0.23*	*1.74*	*1.12*	*2.72*	*0.014*
	Lawful Shooting	Don’t Know	Linear	−0.49	0.17	0.61	0.44	0.85	0.003
		*Incorrect*	*Linear*	*−0.68*	*0.17*	*0.51*	*0.37*	*0.70*	*<0.001*
	Fear In Home	Don’t Know	Linear	−0.68	0.15	0.51	0.37	0.68	<0.001
		Don’t Know	4th effect	0.34	0.14	1.40	1.06	1.85	0.017
		*Incorrect*	*Linear*	*−1.04*	*0.16*	*0.36*	*0.26*	*0.49*	*<0.001*
Nation-specific Laws	Licencing	Don’t Know	Linear	−1.26	0.21	0.28	0.19	0.43	<0.001
		*Incorrect*	*Quadratic*	*0.78*	*0.15*	*2.18*	*1.62*	*2.94*	*<0.001*
		*Incorrect*	*4th effect*	*−0.31*	*0.14*	*0.73*	*0.55*	*0.97*	*0.031*
	Leads Around Livestock	Don’t Know	Linear	−0.80	0.20	0.45	0.31	0.66	<0.001
		*Incorrect*	*Linear*	*0.53*	*0.17*	*1.70*	*1.22*	*2.38*	*0.002*
	Welfare Transfer	*Incorrect*	*5th effect*	*−0.42*	*0.21*	*0.66*	*0.44*	*0.99*	*0.045*
Hypothetical Laws	Public Access	Don’t Know	Linear	−1.07	0.19	0.34	0.24	0.50	<0.001
		*Incorrect*	*Linear*	*−1.80*	*0.23*	*0.17*	*0.11*	*0.26*	*<0.001*
	Restricted Neutering	Don’t Know	Linear	−1.06	0.18	0.35	0.24	0.49	<0.001
		Don’t Know	Quadratic	0.39	0.17	1.48	1.05	2.07	0.024
		*Incorrect*	*Linear*	*−1.44*	*0.21*	*0.24*	*0.16*	*0.36*	*<0.001*
	Mandatory Exercise	Don’t Know	Linear	−0.78	0.17	0.46	0.33	0.64	<0.001
		*Incorrect*	*Linear*	*−1.42*	*0.19*	*0.24*	*0.17*	*0.35*	*<0.001*
	Dogs In Rentals	Don’t Know	Linear	−0.71	0.18	0.49	0.35	0.69	<0.001
		*Incorrect*	*Linear*	*−1.18*	*0.18*	*0.31*	*0.22*	*0.44*	*<0.001*
	Left Alone Limit	*Incorrect*	*Linear*	*−0.89*	*0.18*	*0.41*	*0.29*	*0.58*	*<0.001*
	Import Mutilation Ban	*Incorrect*	*Linear*	*1.04*	*0.19*	*2.83*	*1.94*	*4.11*	*<0.001*
	Prong Collar Ban	Don’t Know	Linear	0.87	0.22	2.38	1.53	3.69	<0.001
		*Incorrect*	*Linear*	*1.35*	*0.21*	*3.84*	*2.56*	*5.76*	*<0.001*
	Aversive Training Ban	*Incorrect*	*Linear*	*1.37*	*0.31*	*3.94*	*2.14*	*7.23*	*<0.001*

**Table 8:** Significant results from multinomial models showed that Income primarily had a non-linear effect on knowledge for hypothetical laws. Correct was the baseline category and so it is not shown in the table. Adjusted odds ratios (AORs) are calculated by exponentiating the Estimate. SE is the Standard Error, LL is the lower limit, and UL is the upper limit of the 95% Confidence Interval (CI). Income was modelled as an ordered factor using polynomial contrasts, and only significant contrasts are reported. For linear effects, adjusted odds ratios (AORs) greater than one indicate that as income increases, the odds of the responses increase, while AORs less than one indicate decreased odds. For quadratic effects, AORs greater than one indicate a positive quadratic effect (u-shaped curve) and less than one indicate a negative quadratic effect. Please see File S7 for all results for each polynomial orthogonal contrast including non-significant results. Incorrect responses are italicised to aid comparison.

Status	Item	Response	Contrast	Estimate	SE	AOR	95% CI		*p*
							*LL*	*UL*	
Current Laws	Lawful Shooting	Don’t Know	Linear	−1.25	0.37	0.29	0.14	0.60	<0.001
		Don’t Know	Quadratic	−1.20	0.36	0.30	0.15	0.60	<0.001
		Don’t Know	Cubic	−0.69	0.31	0.50	0.27	0.92	0.027
		Don’t Know	6th effect	−0.42	0.19	0.65	0.46	0.94	0.022
		*Incorrect*	*Quadratic*	*−0.62*	*0.25*	*0.54*	*0.33*	*0.87*	*0.012*
Hypothetical Laws	Public Access	Don’t Know	Linear	−1.18	0.44	0.31	0.13	0.73	0.008
		Don’t Know	6th effect	−0.47	0.21	0.63	0.41	0.95	0.028
		*Incorrect*	*Quadratic*	*0.73*	*0.24*	*2.07*	*1.29*	*3.33*	*0.003*
	Mandatory Exercise	Don’t Know	Linear	−0.68	0.30	0.51	0.28	0.90	0.021
		*Incorrect*	*Quadratic*	*0.76*	*0.24*	*2.13*	*1.34*	*3.39*	*0.001*
	Dogs In Rentals	*Incorrect*	*Quadratic*	*0.65*	*0.23*	*1.92*	*1.23*	*3.00*	*0.004*
		*Incorrect*	*Cubic*	*0.59*	*0.22*	*1.81*	*1.16*	*2.80*	*0.008*
	Brachycephalic Breeding Ban	Don’t Know	Linear	−0.90	0.27	0.41	0.24	0.69	<0.001
		*Incorrect*	*Quadratic*	*0.59*	*0.23*	*1.81*	*1.15*	*2.86*	*0.011*
		*Incorrect*	*7th effect*	*0.40*	*0.16*	*1.50*	*1.09*	*2.05*	*0.012*
	Left Alone Limit	Don’t Know	Linear	−0.83	0.28	0.44	0.25	0.76	0.004
		Don’t Know	5th effect	0.40	0.20	1.49	1.01	2.19	0.044
		*Incorrect*	*Quadratic*	*0.50*	*0.23*	*1.65*	*1.06*	*2.57*	*0.028*

**Table 9:** Significant results from multinomial models showed that Education primarily had a non-linear effect on knowledge. Correct was the baseline and so it is not shown in the table. Adjusted odds ratios (AORs) are calculated by exponentiating the Estimate. SE is the Standard Error, LL is the lower limit, and UL is the upper limit of the 95% Confidence Interval (CI). Education was modelled as an ordered factor using polynomial contrasts, and only significant contrasts are reported. For linear effects, adjusted odds ratios (AORs) greater than one indicate that as educational attainment increases, the odds of the responses increase, while AORs less than one indicate decreased odds. For quadratic effects, AORs greater than one indicate a positive quadratic effect (u-shaped curve) and less than one indicate negative quadratic effects. Please see File S7 for all results for each polynomial orthogonal contrast including non-significant results. Incorrect responses are italicised to aid comparison.

Status	Item	Response	Contrast	Estimate	SE	AOR	95% CI		*p*
							*LL*	*UL*	
Current Laws	Abandonment Ban	*Incorrect*	*4th effect*	*−0.67*	*0.33*	*0.51*	*0.27*	*0.97*	*0.040*
	Ear Cropping Ban	Don’t Know	Quadratic	0.66	0.26	1.94	1.18	3.20	0.009
		Don’t Know	4th effect	−0.41	0.20	0.66	0.45	0.97	0.036
		*Incorrect*	*Linear*	*−0.60*	*0.31*	*0.55*	*0.30*	*1.01*	*0.054*
		*Incorrect*	*Quadratic*	*1.25*	*0.32*	*3.48*	*1.86*	*6.51*	*<0.001*
		*Incorrect*	*Cubic*	*−0.45*	*0.21*	*0.64*	*0.42*	*0.96*	*0.031*
Nation-Specific Laws	Licencing	Don’t Know	Quadratic	0.71	0.28	2.02	1.17	3.51	0.012
		*Incorrect*	*Linear*	*0.66*	*0.29*	*1.93*	*1.10*	*3.39*	*0.022*
		*Incorrect*	*4th effect*	*−0.35*	*0.17*	*0.70*	*0.50*	*0.98*	*0.037*
Hypothetical Laws	Restricted Neutering	*Incorrect*	*Quadratic*	*0.68*	*0.32*	*1.98*	*1.05*	*3.71*	*0.034*
	Mandatory Exercise	Don’t Know	Linear	−0.61	0.27	0.55	0.32	0.94	0.028
		*Incorrect*	*Linear*	*−0.74*	*0.29*	*0.48*	*0.27*	*0.85*	*0.011*
		*Incorrect*	*Quadratic*	*0.59*	*0.27*	*1.80*	*1.07*	*3.04*	*0.027*
		*Incorrect*	*4th effect*	*0.47*	*0.19*	*1.60*	*1.10*	*2.33*	*0.014*
	Dogs In Rentals	Don’t Know	Linear	−0.93	0.28	0.39	0.23	0.69	0.001
		Don’t Know	Quadratic	0.54	0.26	1.72	1.04	2.85	0.035
		Don’t Know	4th effect	0.41	0.17	1.51	1.07	2.12	0.019
		*Incorrect*	*Linear*	*−0.75*	*0.28*	*0.47*	*0.27*	*0.82*	*0.008*
		*Incorrect*	*Quadratic*	*0.86*	*0.26*	*2.37*	*1.42*	*3.96*	*<0.001*
	Brachycephalic Breeding Ban	Don’t Know	4th effect	0.61	0.16	1.83	1.35	2.50	<0.001
	Left Alone Limit	*Incorrect*	*Linear*	*−0.75*	*0.29*	*0.47*	*0.27*	*0.84*	*0.011*
		*Incorrect*	*4th effect*	*0.73*	*0.18*	*2.08*	*1.45*	*2.96*	*<0.001*

**Table 10:** Significant results from multinomial models showed that Northern Irish participants tended to answer the most accurately of the four nations. Correct was the baseline and so it is not shown in the table. Incorrect responses are italicised to aid comparison. Adjusted odds ratios (AOR) are calculated by exponentiating the Estimate. SE is the Standard Error, LL is the lower limit, and UL is the upper limit of the 95% Confidence Interval (CI). Adjusted odds ratios (AORs) greater than one indicate increased odds of the response among Northern Irish (NI), Scottish, or Welsh participants compared to English participants, while those less than one indicate reduced odds. Lines highlighted in blue indicate the nation where the law is in force for nation-specific items. Please see File S7 for all results including non-significant results.

Status	Item	Response	Nation	Estimate	SE	AOR	95% CI		*p*
							*LL*	*UL*	
Current Laws	Fouling Fines	Don’t Know	NI	−0.60	0.31	0.55	0.30	1.01	0.053
	Collar ID	*Incorrect*	*Scotland*	*0.49*	*0.15*	*1.64*	*1.22*	*2.21*	*0.001*
	Lawful Shooting	Don’t Know	Scotland	−0.41	0.17	0.66	0.48	0.92	0.015
		*Incorrect*	*NI*	*−0.39*	*0.19*	*0.68*	*0.47*	*0.97*	*0.035*
Nation-Specific Laws	Licencing	Don’t Know	NI	−0.67	0.23	0.51	0.32	0.81	0.004
		Don’t Know	Scotland	−0.51	0.22	0.60	0.39	0.93	0.021
		*Incorrect*	*NI*	*−1.50*	*0.19*	*0.22*	*0.15*	*0.33*	*<0.001*
		*Incorrect*	*Scotland*	*−0.67*	*0.15*	*0.51*	*0.38*	*0.69*	*<0.001*
		*Incorrect*	*Wales*	*−0.32*	*0.15*	*0.73*	*0.54*	*0.98*	*0.036*
	Shock Collar Ban	Don’t Know	Wales	−1.88	0.22	0.15	0.10	0.23	<0.001
		*Incorrect*	*NI*	*−0.56*	*0.20*	*0.57*	*0.38*	*0.85*	*0.005*
		*Incorrect*	*Wales*	*−3.83*	*0.22*	*0.02*	*0.01*	*0.03*	*<0.001*
	Leads Around Livestock	Don’t Know	NI	−1.33	0.21	0.26	0.18	0.40	<0.001
		*Incorrect*	*NI*	*−2.48*	*0.19*	*0.08*	*0.06*	*0.12*	*<0.001*
	Welfare Transfer	Don’t Know	Scotland	−2.04	0.23	0.13	0.08	0.20	<0.001
		*Incorrect*	*NI*	*0.63*	*0.30*	*1.87*	*1.05*	*3.35*	*0.033*
		*Incorrect*	*Scotland*	*−4.90*	*0.28*	*0.01*	*0.00*	*0.01*	*<0.001*
Hypothetical Laws	Restricted Neutering	*Incorrect*	*NI*	*−0.98*	*0.23*	*0.37*	*0.24*	*0.58*	*<0.001*
		*Incorrect*	*Scotland*	*−0.49*	*0.20*	*0.62*	*0.42*	*0.91*	*0.014*
	Mandatory Exercise	Don’t Know	Scotland	0.39	0.17	1.48	1.07	2.05	0.019
	Dogs In Rentals	Incorrect	NI	−0.70	0.19	0.50	0.34	0.73	<0.001
	Prong Collar Ban	Don’t Know	Wales	0.54	0.25	1.71	1.05	2.80	0.032

**Table 11:** Comparison of participants’ responses to nation-specific laws by UK Nation of residence showed that participants generally answered items similarly regardless of whether the law was in force in their respective nations, with the exception of Licencing. Lines highlighted in blue indicate the nation where the law is in force for these nation-specific items.

Item	UK Nation	True	Don’t Know	False
Shock Collar Ban	England	71% (351)	17% (85)	12% (59)
	Northern Ireland	57% (204)	25% (89)	19% (67)
	Scotland	73% (326)	14% (62)	13% (58)
	Wales	75% (333)	16% (71)	9% (41)
Welfare Transfer	England	74% (365)	18% (88)	8% (42)
	Northern Ireland	79% (284)	16% (58)	5% (18)
	Scotland	74% (332)	21% (92)	5% (22)
	Wales	76% (336)	17% (75)	8% (34)
Licencing	England	38% (189)	14% (67)	48% (239)
	Northern Ireland	76% (274)	11% (40)	13% (46)
	Scotland	27% (122)	11% (50)	61% (274)
	Wales	30% (133)	14% (61)	56% (251)
Leads Around Livestock	England	64% (316)	21% (103)	15% (76)
	Northern Ireland	59% (212)	21% (75)	20% (73)
	Scotland	66% (294)	18% (79)	16% (73)
	Wales	62% (277)	20% (87)	18% (81)

**Table 12:** Exploratory results from multinomial models showed that Northern Irish participants answered with the most accuracy (despite still being mostly incorrect). True was the baseline and so it is not shown in the table. ‘Don’t Know’ responses are italicised to aid comparison. Adjusted odds ratios (AORs) are calculated by exponentiating the Estimate. SE is the Standard Error, LL is the lower limit, and UL is the upper limit of the 95% Confidence Interval (CI). Adjusted odds ratios (AORs) greater than one indicate increased odds of the response among Northern Irish, Scottish, or Welsh participants compared to English participants, while those less than one indicate reduced odds. Licencing is only in force in Northern Ireland, Shock Collar Ban is only in force in Wales, and Welfare Transfer is only in force in Scotland. Please see File S7 for all results including non-significant results.

Item	Response	UK Nation	Estimate	SE	AOR	95% CI		*p*
						*LL*	*UL*	
Licencing (Northern Ireland)	False	Northern Ireland	−2.02	0.19	0.13	0.09	0.19	<0.005
	False	Scotland	0.57	0.15	1.78	1.33	2.36	<0.005
	False	Wales	0.40	0.14	1.49	1.12	1.98	0.006
	*Don’t Know*	*Northern Ireland*	*−0.89*	*0.22*	*0.41*	*0.27*	*0.64*	*<0.005*
Shock Collar Ban (Wales)	False	Northern Ireland	0.67	0.20	1.95	1.32	2.89	<0.005
	*Don’t Know*	*Northern Ireland*	*0.59*	*0.18*	*1.80*	*1.28*	*2.54*	*<0.005*
Welfare Transfer (Scotland)	False	Northern Ireland	−0.60	0.29	0.55	0.31	0.98	0.042
	False	Scotland	−0.55	0.27	0.58	0.34	0.98	0.044

**Table 13:** Significant results from multinomial models showed that Urbanicity was rarely significantly associated with legal knowledge, but when it was, it was only significantly associated for current and nation-specific laws. Correct was the baseline and so it is not shown in the table. Adjusted odds ratios (AORs) are calculated by exponentiating the Estimate. SE is the Standard Error, LL is the lower limit, and UL is the upper limit of the 95% Confidence Interval (CI). Bolding signifies significant results at *p* < 0.05. The item name is bolded when both responses are significant. Incorrect responses are italicised to aid comparison. Adjusted odds ratios (AORs) greater than one indicate that as Urbanicity increases (postcode districts have a higher percentage of urban areas), there are increased odds of the responses, while AORs less than one indicate reduced odds of responses. Please see File S7 for all results including non-significant ones.

Status	Item	Response	Estimate	SE	AOR	95% CI		*p*
						*LL*	*UL*	
Current Laws	Fouling Fines	**Don’t Know**	**−0.63**	**0.31**	**0.53**	**0.29**	**0.97**	**0.040**
		*Incorrect*	*0.34*	*0.37*	*1.41*	*0.68*	*2.91*	*0.354*
	Lawful Shooting	**Don’t Know**	**0.77**	**0.23**	**2.17**	**1.39**	**3.38**	**<0.001**
		*Incorrect*	*−0.01*	*0.21*	*0.99*	*0.66*	*1.49*	*0.969*
Nation-Specific Laws	Licencing	Don’t Know	−0.24	0.27	0.79	0.47	1.33	0.377
		***Incorrect***	***0.44***	***0.21***	***1.56***	***1.04***	***2.43***	***0.031***
	Shock Collar Ban	Don’t Know	0.05	0.25	1.05	0.64	1.72	0.841
		***Incorrect***	***0.55***	***0.23***	***1.73***	***1.10***	***2.72***	***0.018***

**Table 14:** Significant results from multinomial models showed that Never Owners were always at significantly greater odds of selecting ‘Don’t Know’ compared to current dog owners, and in some cases were more likely to respond incorrectly to nation-specific and hypothetical laws. The reference category that Never Owners are compared to could be First-Time Owners and Current Owners which combined First-Time Owners and Longtime Owners. Items with a ^1^ indicate that the model used Dog History with the reference category as First-Time Owners with Longtime Owners as a separate category. All other items used Current Owners as the reference category which combined First-Time Owners and Longtime Owners. When Current Owners were used as the reference category, Longtime Owners did not differ significantly and were combined with First-Time Owners to improve model performance. Correct was the baseline for the outcome variable and so it is not shown in the table. Incorrect responses are italicised to help differentiate from ‘Don’t Know’ results. Adjusted odds ratios (AORs) are calculated by exponentiating the Estimate. SE is the Standard Error, LL is the lower limit, and UL is the upper limit of the 95% Confidence Interval (CI). Adjusted odds ratios (AORs) greater than one indicate increased odds of the response among Never Owners compared to either Current or First-Time Owners while AORs less than one indicate reduced odds. Please see File S7 for all results including non-significant ones.

Status	Item	Response	Estimate	SE	AOR	95% CI		*p*
						*LL*	*UL*	
Current Laws	Restricted Breeds	Don’t Know	0.79	0.20	2.20	1.49	3.23	<0.001
	Fouling Fines	Don’t Know	1.00	0.21	2.73	1.79	4.15	<0.001
	Abandonment Ban	Don’t Know	0.74	0.16	2.10	1.54	2.87	<0.001
	Ear Cropping Ban	Don’t Know	1.00	0.14	2.71	2.05	3.58	<0.001
	Straying Ban	Don’t Know	0.80	0.13	2.23	1.72	2.89	<0.001
	Collar ID	Don’t Know	1.03	0.15	2.81	2.11	3.75	<0.001
	Lawful Shooting ^1^	Don’t Know	0.54	0.14	1.72	1.30	2.27	<0.001
	Fear In Home ^1^	Don’t Know	0.59	0.13	1.80	1.38	2.33	<0.001
Nation-specific Laws	Licencing	Don’t Know	1.32	0.18	3.74	2.63	5.34	<0.001
		*Incorrect*	*0.92*	*0.14*	*2.52*	*1.92*	*3.29*	*<0.001*
	Shock Collar Ban	Don’t Know	0.67	0.18	1.95	1.37	2.77	<0.001
		*Incorrect*	*0.32*	*0.16*	*1.38*	*1.01*	*1.89*	*0.042*
	Leads Around Livestock	Don’t Know	0.56	0.17	1.75	1.26	2.43	<0.001
	Welfare Transfer	Don’t Know	0.88	0.19	2.40	1.64	3.52	<0.001
Hypothetical Laws	Public Access	Don’t Know	0.60	0.16	1.81	1.33	2.47	<0.001
	Restricted Neutering ^1^	Don’t Know	1.47	0.17	4.34	3.10	6.08	<0.001
	Mandatory Exercise	Don’t Know	0.72	0.14	2.04	1.55	2.70	<0.001
		*Incorrect*	*0.32*	*0.16*	*1.38*	*1.01*	*1.90*	*0.044*
	Dogs In Rentals	Don’t Know	0.71	0.15	2.03	1.51	2.72	<0.001
	Brachycephalic Breeding Ban ^1^	Don’t Know	0.61	0.13	1.84	1.41	2.39	<0.001
		*Incorrect*	*0.33*	*0.15*	*1.39*	*1.04*	*1.86*	*0.028*
	Left Alone Limit	Don’t Know	1.16	0.14	3.19	2.42	4.20	<0.001
		*Incorrect*	*0.39*	*0.15*	*1.47*	*1.09*	*1.99*	*0.012*
	Restricted Euthanasia	Don’t Know	1.21	0.19	3.36	2.33	4.84	<0.001
		*Incorrect*	*0.49*	*0.16*	*1.64*	*1.19*	*2.26*	*0.003*
	Import Mutilation Ban	Don’t Know	0.81	0.18	2.24	1.58	3.18	<0.001
	Prong Collar Ban	Don’t Know	0.97	0.20	2.64	1.78	3.92	<0.001
	Aversive Training Ban	Don’t Know	0.68	0.30	1.98	1.09	3.59	0.025

**Table 15:** Significant results from multinomial models showed that Past Owners tended to be more uncertain than current dog owners but for fewer items than Never Owners and to a lesser extent. The reference category that Never Owners are compared to could be First-Time Owners or Current Owners which combined First-Time Owners and Longtime Owners. Items with a ^1^ indicate that the model used Dog History with the reference category as First-Time Owners with Longtime Owners as a separate category. All other items used Current Owners as the reference category which combined First-Time Owners and Longtime Owners. When Current Owners were used as the reference category, Longtime Owners did not differ significantly and were combined with First-Time Owners to improve model performance. Correct was the baseline for the outcome variable and so it is not shown in the table. Incorrect responses are italicised to help differentiate from ‘Don’t Know’ results. Adjusted odds ratios (AORs) are calculated by exponentiating the Estimate. SE is the Standard Error, LL is the lower limit, and UL is the upper limit of the 95% Confidence Interval (CI). Adjusted odds ratios (AORs) greater than one indicate increased odds of the response among Past Owners compared to either Current or First-Time Owners, while AORs less than one indicate reduced odds. Please see File S7 for all results including non-significant ones.

Status	Item	Response	Estimate	SE	AOR	95% CI		*p*
						*LL*	*UL*	
Current Laws	Restricted Breeds	Don’t Know	0.71	0.26	2.04	1.23	3.38	0.006
	Fouling Fines	Don’t Know	0.63	0.29	1.88	1.06	3.32	0.030
	Ear Cropping Ban	Don’t Know	0.37	0.19	1.45	1.00	2.08	0.047
		*Incorrect*	*−0.67*	*0.32*	*0.51*	*0.27*	*0.97*	*0.039*
	Fear In Home ^1^	Don’t Know	0.40	0.16	1.49	1.09	2.05	0.014
Nation-Specific Laws	Licencing	Don’t Know	0.73	0.25	2.08	1.29	3.36	0.003
		*Incorrect*	*0.74*	*0.16*	*2.10*	*1.52*	*2.90*	*<0.001*
	Shock Collar Ban	Don’t Know	0.54	0.21	1.72	1.13	2.60	0.011
		*Incorrect*	*0.36*	*0.19*	*1.44*	*0.99*	*2.09*	*0.057*
	Leads Around Livestock	Don’t Know	0.42	0.22	1.52	0.99	2.34	0.056
Hypothetical Laws	Public Access	Don’t Know	0.43	0.20	1.53	1.03	2.28	0.036
	Restricted Neutering ^1^	Don’t Know	0.83	0.22	2.29	1.50	3.50	<0.001
	Mandatory Exercise	Don’t Know	0.63	0.17	1.89	1.34	2.65	<0.001
	Left Alone Limit	Don’t Know	0.60	0.17	1.83	1.32	2.53	<0.001

**Table 16:** Significant results from multinomial models showed that Longtime Owners rarely significantly differed from First-Time Owners. When they did, they answered more accurately for hypothetical laws and less accurately for current laws. Correct was the baseline for the outcome variable and so it is not shown in the table. Incorrect responses are italicised to help differentiate from ‘Don’t Know’ results. Adjusted odds ratios (AORs) are calculated by exponentiating the Estimate. SE is the Standard Error, LL is the lower limit, and UL is the upper limit of the 95% Confidence Interval (CI). Adjusted odds ratios (AORs) greater than one indicate increased odds of the responses among Longtime Owners compared to First-Time Owners, while those AORs of less than one indicate reduced odds. Please see File S7 for all results including non-significant ones.

Status	Item	Response	Estimate	SE	AOR	95% CI		*p*
						*LL*	*UL*	
Current Laws	Lawful Shooting	*Incorrect*	*0.53*	*0.27*	*1.70*	*1.01*	*2.87*	*0.047*
	Fear In Home	*Incorrect*	*0.85*	*0.27*	*2.34*	*1.39*	*3.95*	*0.001*
Hypothetical Laws	Restricted Neutering	*Incorrect*	*−1.15*	*0.55*	*0.32*	*0.11*	*0.93*	*0.036*
	Brachycephalic Breeding Ban	Don’t Know	−0.66	0.31	0.51	0.28	0.94	0.030
		*Incorrect*	*−0.95*	*0.37*	*0.39*	*0.19*	*0.81*	*0.011*

**Table 17:** Results from multinomial models showed that those with a childhood dog often answered with more certainty and accuracy than those who did not have a dog as a child, particularly for hypothetical laws. Correct was the baseline and so it is not shown in the table. Incorrect responses are italicised to help differentiate from ‘Don’t Know’ results. Adjusted odds ratios (AORs) is calculated by exponentiating the Estimate. SE is the Standard Error, LL is the lower limit, and UL is the upper limit of the 95% Confidence Interval (CI). Adjusted odds ratios (AORs) greater than one indicate increased odds of the response among those who had a childhood dog compared to those who did not have a childhood dog, while those less than one indicate reduced odds. Bolding signifies significant results at *p* < 0.05. The item name is bolded when both responses are significant. Please see File S7 for all results including non-significant ones.

Status	Item	Response	Estimate	SE	AOR	95% CI		*p*
						*LL*	*UL*	
Current Laws	Fouling Fines	**Don’t Know**	**−0.96**	**0.28**	**0.38**	**0.22**	**0.66**	**<0.001**
		*Incorrect*	*0.16*	*0.23*	*1.18*	*0.75*	*1.86*	*0.479*
	Straying Ban	**Don’t Know**	**−0.28**	**0.14**	**0.76**	**0.58**	**0.99**	**0.043**
		*Incorrect*	*−0.04*	*0.16*	*0.96*	*0.71*	*1.30*	*0.801*
	Ear Cropping Ban	**Don’t Know**	**−0.39**	**0.15**	**0.68**	**0.51**	**0.91**	**0.011**
		*Incorrect*	*−0.19*	*0.24*	*0.83*	*0.52*	*1.33*	*0.434*
Nation-Specific Laws	Licencing	**Don’t Know**	**−0.48**	**0.20**	**0.62**	**0.42**	**0.92**	**0.016**
		*Incorrect*	*−0.20*	*0.14*	*0.82*	*0.62*	*1.07*	*0.138*
Hypothetical Laws	**Public Access**	**Don’t Know**	**−0.50**	**0.17**	**0.61**	**0.43**	**0.85**	**0.004**
		***Incorrect***	***−0.70***	***0.22***	***0.50***	***0.32***	***0.76***	***0.001***
	Restricted Neutering	**Don’t Know**	**−0.44**	**0.17**	**0.65**	**0.46**	**0.91**	**0.013**
		*Incorrect*	*−0.33*	*0.20*	*0.72*	*0.48*	*1.06*	*0.098*
	**Mandatory Exercise**	**Don’t Know**	**−0.41**	**0.15**	**0.66**	**0.50**	**0.88**	**0.005**
		***Incorrect***	***−0.44***	***0.17***	***0.65***	***0.46***	***0.91***	***0.013***
	**Dogs In Rentals**	**Don’t Know**	**−0.42**	**0.16**	**0.66**	**0.48**	**0.89**	**0.006**
		***Incorrect***	***−0.47***	***0.16***	***0.63***	***0.46***	***0.87***	***0.005***
	**Left Alone Limit**	**Don’t Know**	**−0.28**	**0.14**	**0.75**	**0.58**	**0.99**	**0.040**
		***Incorrect***	***−0.39***	***0.16***	***0.68***	***0.50***	***0.92***	***0.012***

## Data Availability

The original contributions presented in this study are included in the article/Supplementary Material. Further inquiries can be directed to the corresponding author.

## Supplementary Materials

The following supporting information can be downloaded at: https://www.mdpi.com/article/10.3390/ani16101463/s1, File S1: Pattern Analysis of Missing Data; File S2: Survey; File S3: Urbanicity Calculation and Data for Northern Irish and Scottish Postcode Districts; File S4: Item Development; File S5: Code for Data Preparation and Calculating Urbanicity; File S6: Code for Model Building and Data Analysis; File S7: Full Results, Model Building Results and Model Diagnostics; File S8: Participant Data, Data Quality Checks and Nat Rep Quotas. References [112,113,114,115,116,117,118,119] are cited in the Supplementary Materials.

## Abbreviations

The following abbreviations are used in this manuscript:

UK	United Kingdom
AOR	Adjusted Odds Ratio

## Appendix A. Development of Urbanicity

Definitions of whether an area is urban or rural differ across the UK. This is a reflection of the differences in the nature of rurality across the UK resulting from the variations in the geography, population size, density and distribution across the four nations [106] (see Table A1). There are separate governmental classification systems in Northern Ireland, Scotland, and a shared system for England and Wales that take these factors into account. Currently, to our knowledge, there is no single standardised, peer reviewed method to classify urban and rural areas across the UK as a whole.

**Table A1:** The UK has diverse geographies and populations, that results in different approaches to classifying urban and rural areas. ‘Area % rural’ is the percentage of land in each nation classified as rural. ‘% Population Urban’ is the percentage of the population in each UK Nation that lives in an urban area. Urbanicity Average is the average urbanicity score derived for each nation in the current study (details below).

UK Nation	Area (km^2^)	Area % Rural	Population (Millions)	% Population Urban	Urbanicity Average
England	132,930	85%	58.6	83%	81%
Northern Ireland	14,330	46%	1.9	56%	70%
Scotland	80,231	98%	5.5	83%	83%
Wales	21,218	82%	3.1	68%	70%

Sources: [104,182,183,184,185,186,187].

The lack of a standardised UK-wide classification system is largely a result of the very different government statistical systems used to classify urban and rural areas in the UK. All systems use settlements (Northern Ireland and Scotland) or Built-up Areas (England and Wales) as the basis of their classifications systems, which were developed using different methods reflecting the distinct priorities of each nation (see Table A2) [188,189,190]. Therefore, the characteristics of a settlement in Scotland may differ substantially from those of a Built-up Area in England. A single standardised classification would risk misclassifying participants. For example, a hypothetical small town of around 5000 people in Northern Ireland may operate as a local hub with shops, healthcare services, and other amenities that serve surrounding rural communities. Whereas in England, a Built-up Area of a similar size may function more like a rural area located within commuting distance of a larger urban centre. We therefore used each UK nation’s own rural/urban classification system to develop an Urbanicity score to more meaningfully categorise participants within their national context.

**Table A2:** Each nation defines Built-up Areas or settlements differently. These settlement definitions form the foundational geographic units on which each nation’s rural/urban classification systems are constructed.

Nation	Definition	Source
England and Wales	Land which is ‘irreversibly urban in character’. Include areas of built-up land with a minimum of 200,000 m^2^	[189]
Northern Ireland	Defined by Department of Environment and local council planning agencies	[188,191]
Scotland	A group of high-density postcodes whose combined population rounds up to 500 people or more	[190]

### Appendix A.1. The UK’s Postcode System

We used the UK’s postcode system, maintained by the Royal Mail [192] in conjunction with the governmental definitions relevant to each nation, to calculate an Urbanicity score. Postcodes have a common structure across the UK (see Table A3). While England, Scotland and Wales have a number of different Postcode Areas, Northern Ireland only has one. All Northern Irish Postcodes start with the area ‘BT’. We only collected participants’ Postcode District rather than full Unit Postcode to maintain participant anonymity and calculated an Urbanicity score for each participants’ Postcode District.

**Table A3:** The structure of all UK postcodes reproduced from Office for National Statistics [192]. All parts of the postcode, even those which were not used in the current study, are shown for context.

Example	Geographic Unit	Number of Geographic Units in the UK
PO	Postcode Area	124
PO15	Postcode District	3118
PO15 5	Postcode Sector	12,463
PO15 5RR	Unit Postcode	Approximately 1.79 million (Live)

### Appendix A.2. Calculating Urbanicity

To create a measure of the degree to which a postcode district is urban, we calculated the proportion of postcodes classified as urban within each postcode district relative to the total number of postcodes in that district. Urbanicity was calculated separately for each nation’s classification system according to the available data and the definitions used within each system.

(A1) $$Urbanicity=\frac{Number\;of\;urban\;postcodes\;in\;a\;postcode\;district}{Total\;number\;of\;postcodes\;in\;a\;postcode\;district}$$

### Appendix A.3. Calculating Urbanicity for Each Nation

Although each nation has more complex, in-depth classification systems, we used their binary (rural/urban) versions to aid comparisons (See [104,184,190] for detailed overviews of each system’s classification system). See Table A4 for a comparison of the different classification systems.

**Table A4:** Overview of each national governmental classification system. Although the binary systems use population size as a form of threshold, the underlying logic and application of these thresholds differ reflecting the population size, distribution and density of the nations. The more detailed versions of the classification systems (categories, bands and folds) that correspond to urban and rural are included in brackets.

Nation	Urban	Rural
England and Wales	Output areas that are within a Built-up Area that has a population of over 10,000 people (Categories A1–C2)	All other output areas (Categories D1–F2)
Northern Ireland	Settlements with a population of more than 5000 people (Bands A–E)	Settlements with a population less than 5000 people (Bands F–G)
Scotland	All other settlements (Folds 1–5)	All settlements with a population less than 3000 (Folds 6–8)

Sources: [104,105,190,193].

#### Appendix A.3.1. Scotland

We used the two-fold classification system which defines areas as Rural Scotland and Rest of Scotland to enable comparison to the other nations. Areas are defined as Rural Scotland when settlements contain less than 3000 people [190]. All other areas are included as the Rest of Scotland. We included Rural Scotland as Rural and the Rest of Scotland areas as Urban.

Using the Scottish Government’s settlement classifications [190] and the ONS Postcode Directory [194], we created a percentage of the number of postcodes in each postcode district that was designated as Urban out of the total postcodes in that postcode district. For example, the postcode district AB13 has 94 postcodes. 72 postcodes are classified as Urban, and 22 postcodes are classified as Rural. This resulted in AB13 having a Urbanicity score of 77%. See File S3 for the data and File S7 for the code used to calculate Scottish Urbanicity scores.

#### Appendix A.3.2. England and Wales

We used the 2011 rural-urban classification of output areas released in August 2013 [195], which was the most recent version at the time of data analysis. Output areas (the lowest level of geographical area for census statistics) that are within a Built-up Area with a population of more than 10,000 are defined as urban. All other areas are defined as rural [193].

We matched individual postcodes from the ONS Postcode Directory to the postcode districts in the ONS Postcode Directory’s User Guide [193]. Urbanicity was calculated in the same fashion as Scotland. The number of urban postcodes in a postcode district was divided by the total number of postcodes in a postcode district. The data used to calculate English and Welsh Urbanicity scores can be found at ONS [194] and see File S7 for the code used to calculate the scores.

#### Appendix A.3.3. Northern Ireland

Northern Ireland uses a classification system based on the 2011 census [104]. Settlements with a population of more than 5000 people (Bands A–E) are defined as Urban and settlements with populations less than 5000 people (Bands F–G) are defined as Rural.

A paid licence from the Northern Ireland Statistics and Research Agency (NISRA) is required to use Northern Irish postcodes for research purposes, something which was outside of the budget for this project. Therefore, we estimated an Urbanicity score using the settlement data from NISRA [104] which does not include postcodes, but does have the settlement names and electoral wards. These wards represent very small areas that can then be rolled up into Local Government District (LGD) areas which are the basis for most official statistics [104]. All Northern Irish postcodes start with the area code ‘BT’, limiting the number of different postcode districts.

To identify postcode districts that could then be matched to the rural/urban classification, we searched for the 1992 Ward Names with the LGD areas in Google Maps in February 2025. If there were no appropriate matches, then the postcode district that matched the 1992 LGD was used. For example, Oaklands (ward name) in Cookstown (LGA area) did not have suitable results as the only results were in Derry/Londonderry which is far from Cookstown and so is likely to be incorrect. We therefore used the Cookstown postcode district of BT80. Some Google map results returned the main street name in a small town which matched the ward name and LGA. We assumed that the ward name was derived from the major street name or other major landmarks. These assumptions are unlikely to strongly impact results because there are very few small areas impacted. For example, there are four small areas in the ward Oaklands that are rural which means that its inclusion resulted in the Urbanicity score changing from 54% to 52%.

There were two postcode districts, BT1 and BT64, where we found no matches. We assumed BT1 was 100% urban because the area is the city centre of Belfast which is automatically classified as urban. We also assumed BT64 was 100% urban because it is a small area that mostly covers a town centre. This may not be correct, but we had no participants from this postcode district and so this did not impact results. However, others who may use this data should try to validate this postcode.

To create an Urbanicity score for each postcode district, we counted the total number of urban wards in a postcode district and divided by the total number of wards in a postcode district. For example, there are 159 wards assigned to ‘BT23’, with 126 of these classified as urban resulting in an Urbanicity score of 79%. See File S3 for the data and File S7 for the code used to calculate Northern Irish Urbanicity scores.
