# Supplementary material for: Exploring Public Knowledge of Dog Law in the UK: Evidence of Poor Legal Knowledge in a Nationally Representative Sample
Source: Animals (Basel). 2026 May 10;16(10):1463. doi: 10.3390/ani16101463 (PMC13203885; doi:10.3390/ani16101463)
Supplement: Supplementary file 1 [file animals-16-01463-s001.zip › File S1 - Pattern Analysis of Missing Data.pdf]

File S1: Analysis of Patterns of Missing Data

Missing data patterns were examined using a visual aggregation plot produced with the *VIM* package in R (See Figure S1). The plot displays combinations of observed and missing values across key variables (income, UK Nation (country), education, gender, and age), with blue indicating observed data and red indicating missing values. The majority of cases were complete across all variables (approximately 95% of observations). Income accounted for most of the missing data (see Table S1), with 4% of participants selecting 'Prefer not to say' when asked to report their income. Overall, missing data were relatively sparse and largely confined to a small number of cases with few systematic patterns.

Figure S1. Plot created using the VIM package in R shows that we had small numbers of missing data with no evidence of systematic patterns that would influence results. Blue squares indicate complete data and red signifies missing data. Numbers on the right side are the proportion of instances that the combination of red squares occurred. For example, the most frequent combination of missing variables was 0.34% of participants, equating to 7 participants choosing 'prefer not to say' for both Income and Education.

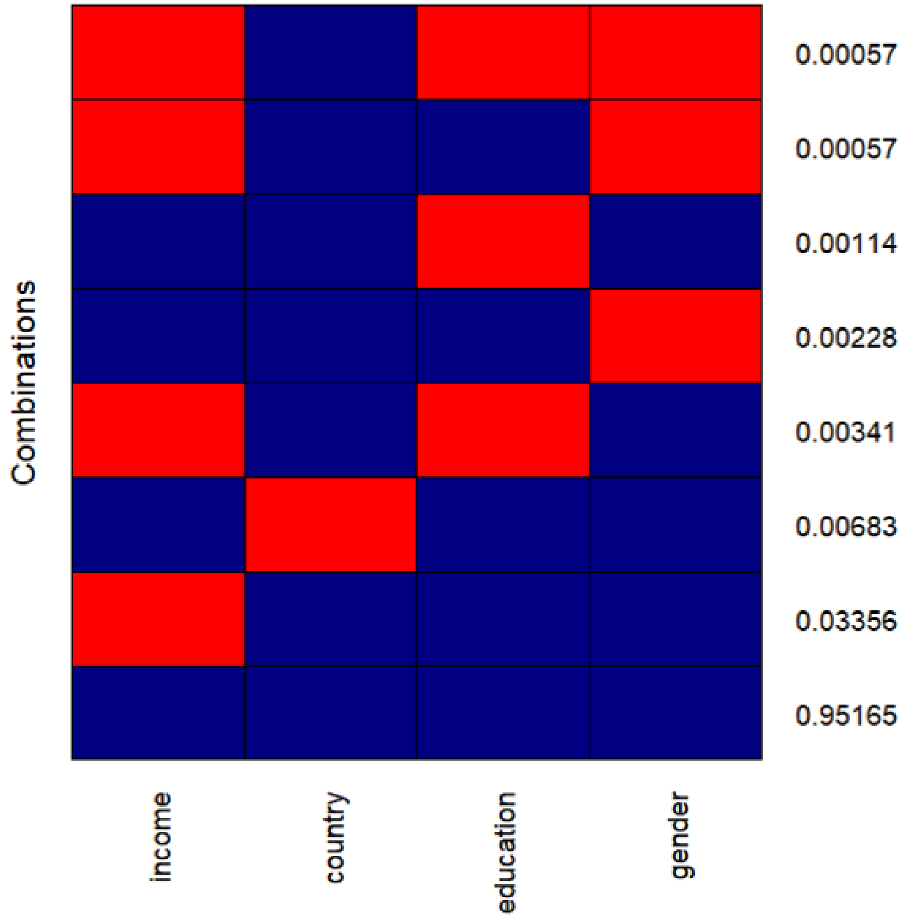

Table S1. Participants were most likely to choose ‘prefer not to say’ for income. Participants were not provided with an option to select ‘prefer not to say’ for Age and Dog History and so there were no missing values for these variables. Missing values for UK Nation was a result of participants’ UK Nation and Postcode District not matching and so were set to missing (Mismatch). Low frequency gender groups were also set to missing.

Variable	Prefer not to say	Mismatch	Low frequency	Total missing	% Missing
Income	67	0	0	67	3.8%
UK Nation	0	12	0	12	0.7%
Education	9	0	0	9	0.5%
Gender	2	0	5	7	0.3%
**Total**	**78**	**12**	**5**	**95**	**5.3%**
