# Supplementary material for: Exploring Public Knowledge of Dog Law in the UK: Evidence of Poor Legal Knowledge in a Nationally Representative Sample
Source: Animals (Basel). 2026 May 10;16(10):1463. doi: 10.3390/ani16101463 (PMC13203885; doi:10.3390/ani16101463)
Supplement: Supplementary file 1 [file animals-16-01463-s001.zip › File S2 - Survey.pdf]

Participant Information Sheet

Please read the following information carefully.

1. Research Project Title:

Evaluating Public Awareness and Attitudes Toward Dog Legislation in Great Britain and Northern Ireland

2. Opening text:

You are invited to participate in a web-based online survey on legislation that impacts dogs or their owners. You have been invited to participate because you live in Northern Ireland, Scotland, England or Wales and are over 18. This survey is part of a research project being conducted by Sarah Weir, a PhD student at the University of Stirling. It should take about 20 minutes to complete. During the survey you will be given some questions to answer about yourself and your knowledge and opinions of dog law. Please read through these terms before agreeing to participate below.

3. Background aims of project

Law applies to everyone in society, governing behaviour in public and private spaces. It creates normative expectations for acceptable behaviour and, when it works well, reduces conflict by balancing the needs of different segments of the population. To be able to achieve these goals, the public must be aware of the law and willing to comply. Dog laws are often enacted at the nation/country level of the UK and so they can vary depending on where someone lives. Thus, it is important to understand the extent to which residents are knowledgeable and supportive of national legislation. Therefore, this study aims to understand the general public's awareness and opinions of different areas of their local dog law.

4. Do I have to take part?

No. Your participation in this survey is voluntary. You may refuse to take part in the research or exit the survey at any time without penalty by pressing the 'Exit' button / closing the browser. Please note that this survey is anonymous.

5. Are there any potential risks in taking part?

There are no foreseeable risks involved in participating in this study.

6. Legal basis for processing personal data

As part of the project we will be recording personal data relating to you. All data will be collected and passed to the research team by Cint. This will be processed in accordance with the General Data Protection Regulations (GDPR). Under GDPR the legal basis for processing your personal data will be public interest/the official authority of the University.

7. What happens to the data I provide?

Your answers will be completely anonymous, and we will use all reasonable endeavours to keep them confidential. All data will be collected by Cint and will be passed to the research team to analyse. Your data will be stored in a password-protected file and may be used in academic publications and conferences, and the findings of the project potentially shared with dog organisations and other stakeholders including the public. Your IP address will not be stored.

The data that we collect from you may be transferred to, and stored or processed at, a destination outside the European Economic Area (EEA) by submitting your personal data, you agree to this transfer, storing or processing.

Your personal data will be kept until data analysis is complete on OneDrive and then will be securely destroyed.

8. Future uses of the data

Due to the nature of this research, it is very likely that other researchers may find the data to be useful in answering other research questions. Before beginning the survey, we will ask for your explicit consent for your data to be shared in this way. Because this study is anonymous, the data will not be able to be traced back to you.

9. Will the research be published?

The results of this study will be submitted in a thesis in part fulfilment of a PhD. The

data from this study may be published in an academic journal, presented at academic conferences and the findings of the project potentially shared with dog organisations to create resources and recommendations to the public. The University of Stirling is committed to making the outputs of research publicly accessible and supports this commitment through our online open access repository STORRE. Unless the publisher requirements prevent us, this research will be publicly disseminated through our open access repository.

10. Who has reviewed this research project?

The ethical approaches of this project have been approved via The University of Stirling's General University Ethics Panel and Animal Welfare and Ethics Review Body. The approval number GUEP 2024 15873 14069.

11. Your rights

You have the right to request to see a copy of the information we hold about you and to request corrections or deletions of the information that is no longer required. You have the right to withdraw from this project at any time without giving reasons and without consequences to you. You also have the right to object to us processing relevant personal data however, please note that once the data are being analysed (1 week after survey completion) it may not be possible to remove your data from the study.

To request a copy of the information we hold about you or to withdraw your data please email Sarah Weir (s.a.weir@stir.ac.uk) with your participant ID given to you at the start of the survey that identifies your anonymous data. Please make sure to take a note of your participant ID in case you wish to do this.

12. Whom do I contact if I have concerns about this study or I wish to complain?

If you would like to discuss the research with someone you can contact the student investigator Sarah Weir (s.a.weir@stir.ac.uk). If you have further questions you can contact the study supervisors Sharon Kessler (sharon.kessler@stir.ac.uk) and Clare Andrews (clare.andrews@stir.ac.uk). If you wish to lodge a complaint you should contact the head of division Professor Paul Dudchenko (p.a.dudchenko@stir.ac.uk).

You have the right to lodge a complaint against the University regarding data protection issues with the Information Commissioner's Office (https://ico.org.uk/concerns/). The University's Data Protection Officer is Joanna Morrow, Deputy Secretary. If you have any questions relating to data protection these can be addressed to data.protection@stir.ac.uk in the first instance.

You may wish to print a copy of this information to keep.

Thank you for your participation!

Next

Consent

This study has been approved by the University of Stirling's ethics committee. The ethics approval number is GUEP 2024 15873 14069.

1. I confirm that I have read and understood the information sheet explaining the research project and I have had the opportunity to ask questions about the project.
2. I understand that my participation is voluntary and that I am free to withdraw at any time during the study and withdraw my data within 1 week of completing the survey without giving a reason, and without any penalty. I understand that beyond 1 week, when data analysis has started it may not be possible to remove my data from the study.
3. I have been given a unique identifying number and know whom to contact should I wish to withdraw my data.
4. I understand that my responses will be kept anonymous and I give permission for members of the research team and Cint to have access to my anonymised responses.
5. I agree for research data collected in the study to be made available to researchers, including those working outside the EU to be used in other research studies. I understand that any data that leave the research group will be fully anonymised so that I cannot be identified.
6. I am 18 or older and a resident of the United Kingdom. I agree to take part in this study.

	I Agree
I have read and understood statements 1-6 and give my consent to participate in the survey	☐

If you do not consent to the terms, please exit your browser or close your session.

Next

For quality purposes, please answer the following questions:

Please select yellow from the list below

- [ ] Red
- [ ] Blue
- [ ] Purple
- [ ] Green
- [ ] Black
- [ ] Yellow
- [ ] Orange
- [ ] Magenta
- [ ] None of the above

A boy had 4 marbles and lost one. How many marbles does he have now?

- [ ] 1
- [ ] 2
- [ ] 3
- [ ] 4
- [ ] 5
- [ ] Not sure
- [ ] None of the above

Next

This section is about your eligibility to take part in the survey

Do you live in Northern Ireland, Scotland, England or Wales?

- ◯ Yes
- ◯ No

Are you aged 18 or over?

- ◯ Yes
- ◯ No

Next

This section includes questions about you

Which country or region do you currently live in?

- Northern Ireland
- Scotland
- Wales
- North East
- North West
- Yorkshire and The Humber
- East Midlands
- West Midlands
- London
- East of England
- South East
- South West

[illegible]

What is your history interacting with and owning dogs? Please select all that apply

- ☐ Currently own at least one dog
- ☐ Look after someone else's dog on a regular basis
- ☐ Previously owned a dog as an adult
- ☐ Lived with a dog but did not own one
- ☐ Had a dog in the household as a child
- ☐ Never owned a dog

What age are you?

- ◯ 18 - 24
- ◯ 25 - 34
- ◯ 35 - 44
- ◯ 45 - 54
- ◯ 55 - 64
- ◯ 65+

What is your gender identity?

- ◯ Male
- ◯ Female
- ◯ Non-binary
- ◯ Prefer not to say
- ◯ Other gender identity - please specify

What is the **first half** of your postcode?

What is your highest level of qualification?

- ◯ University higher degree (e.g. MSc, PhD)
- ◯ First degree level qualification including undergraduate degrees, foundation degrees, graduate membership of a professional institute etc
- ◯ Apprenticeship / Trade school
- ◯ High school/secondary school or equivalent
- ◯ Other school (inc. School leaving exam certificate or matriculation)
- ◯ None of the above
- ◯ Prefer not to say

In which of these brackets does the combined gross (before tax) income of all household members fall?

- ○ Less than £10,000
- ○ £10,001 - £20,000
- ○ £20,001 - £30,000
- ○ £30,001 - £40,000
- ○ £40,001 - £60,000
- ○ £60,001 - £80,000
- ○ £80,001 - £100,000
- ○ £100,001 - £150,000
- ○ £150,001 - £200,000
- ○ Over £200,000
- ○ Prefer not to say

Next

We would like to know your knowledge of dog laws

This section aims to assess your knowledge of dog laws in your local area. We will list various dog laws and ask whether you think they accurately describe the law where you live.

Some statements reflect current UK laws, some are laws in specific parts of the UK, and others are not laws at all.

Please read each statement and indicate whether you believe it is True or False. If you are unsure, please select 'Don't know.'

Certain breeds must be muzzled and on a leash in a public place

- ◯ True
- ◯ False
- ◯ Don't Know

Dogs must at all times wear a collar that bears the name and address of the owner inscribed on a plate, badge or disc

- ◯ True
- ◯ False
- ◯ Don't Know

It is illegal to train animals in a way which inflicts significant pain, distress or harm to the animal

- ◯ True
- ◯ False
- ◯ Don't Know

It is illegal for a dog to be off lead when around livestock

- ◯ True
- ◯ False
- ◯ Don't Know

It is legal for someone to shoot a dog if they are worrying/attacking livestock

- ◯ True
- ◯ False
- ◯ Don't Know

A dog cannot be left alone for more than 6 hours by law

- ◯ True
- ◯ False
- ◯ Don't Know

It is illegal to breed flat faced dog like Pugs and French Bulldogs

- ◯ True
- ◯ False
- ◯ Don't Know

It is illegal to crop a dog's ears

- ◯ True
- ◯ False
- ◯ Don't Know

Any dog who is found to be suffering unnecessarily can be removed by a government authorised person and transferred to another owner without the current owner's consent

- ◯ True
- ◯ False
- ◯ Don't Know

It is illegal to import a dog with cropped ears or a docked tail

- ◯ True
- ◯ False
- ◯ Don't Know

All dog owners must have a license for their dog(s)

- ◯ True
- ◯ False
- ◯ Don't Know

It is unlawful for an owner to abandon their dog

- ◯ True
- ◯ False
- ◯ Don't Know

All owners can legally keep a dog in their rented accommodation

- ◯ True
- ◯ False
- ◯ Don't Know

All dogs have the legal right to enter restaurants, bars and shops with their owners as long as the dog is not a threat to public safety

- ◯ True
- ◯ False
- ◯ Don't Know

It is illegal to use a collar that gives an electric shock to their dog

- ◯ True
- ◯ False
- ◯ Don't Know

It is unlawful for an owner to allow their dog to stray

- ◯ True
- ◯ False
- ◯ Don't Know

Owners can be fined if their dog fouls in a public place

- ◯ True
- ◯ False
- ◯ Don't Know

It is illegal to neuter or spay a dog unless for a health reason

- ◯ True
- ◯ False
- ◯ Don't Know

Dogs should be walked at least every 6 hours by law during the day unless there are welfare reasons

- ◯ True
- ◯ False
- ◯ Don't Know

It is illegal to use a spiked collar (also known as a prong collar)

- ◯ True
- ◯ False
- ◯ Don't Know

Dogs can only be euthanised if there is a reasonable reason to do so

- ◯ True
- ◯ False
- ◯ Don't Know

It is illegal for a dog to cause fear or apprehension in another person in your home or someone else's home

- ◯ True
- ◯ False
- ◯ Don't Know

Note: If you cannot continue to the next page please check you have answered all the questions.

Next

Thank you for participating in this research project

Research Project Title: Evaluating Public Awareness and Attitudes Toward Dog Legislation in Great Britain and Northern Ireland

Background, aims of project

Law applies to everyone in society, governing behaviour in public and private spaces. It creates normative expectations for acceptable behaviour and, when it works well, reduces conflict by balancing the needs of different segments of the population. To be able to achieve these goals, the public must be aware of the law and willing to comply. Dog laws are often enacted at the nation/country level of the UK and so they can vary depending on where someone lives. Thus, it is important to understand the extent to which residents are knowledgeable and supportive of national legislation. Therefore, this study aims to understand the general public's awareness and opinions of different areas of their local dog law.

Additional support

After completing this survey, you may have questions about the law in your local area. To find out more about the laws in your country/nation, contact your local animal welfare organisations. You can contact your local authority / council for rules that govern your local community.

Animal Welfare organisations:

- Dogs Trust (UK Wide)
- The Kennel Club (UK Wide but with a focus on England and Wales)
- Battersea (England and Wales)
- RSPCA (England and Wales)
- USPCA (Northern Ireland)
- SSPCA (Scotland)

Some resources to learn more:

- PDSA dog law guide for owners - link (UK wide)

- Blue Cross dog law guide for owners - link (England and Wales only)
- NI Direct Owning a dog in Northern Ireland - link (Northern Ireland only)
- National Dog Warden Association - link (Scotland only)

To learn more about a dog owner's legal requirements to ensure their dog's welfare, you can access your nation/country's Code of Conduct for Animal Welfare.

- Northern Ireland - link
- Scotland - link
- England and Wales - link

If you wish to withdraw your data

You may withdraw your data by emailing Sarah Weir (s.a.weir@stir.ac.uk) using the unique identifier given to you at the start of the survey. Please note that once the data are being analysed (1 week after survey completion) it may not be possible to remove your data from the study. If you have further questions you can contact the student investigator Sarah Weir (s.a.weir@stir.ac.uk) or the study supervisors Sharon Kessler (sharon.kessler@stir.ac.uk) and Clare Andrews (clare.andrews@stir.ac.uk). If you wish to lodge a complaint you should contact the head of division Professor Paul Dudchenko (p.a.dudchenko@stir.ac.uk).

You have the right to lodge a complaint against the University regarding data protection issues with the Information Commissioner's Office (https://ico.org.uk/concerns/). The University's Data Protection Officer is Joanna Morrow, Deputy Secretary. If you have any questions relating to data protection these can be addressed to data.protection@stir.ac.uk in the first instance.

Once again, I would like to thank you for your participation. Please click next to complete the survey!

Next
